# Selenium intake and multiple health-related outcomes: an umbrella review of meta-analyses

**DOI:** 10.3389/fnut.2023.1263853

**Published:** 2023-09-13

**Authors:** Puze Wang, Bo Chen, Yin Huang, Jin Li, Dehong Cao, Zeyu Chen, Jinze Li, Biao Ran, Jiahao Yang, Ruyi Wang, Qiang Wei, Qiang Dong, Liangren Liu

**Affiliations:** ^1^Department of Urology, West China Hospital, Sichuan University, Chengdu, China; ^2^Department of Urology, Hospital of Chengdu University, Chengdu, China

**Keywords:** selenium, health, umbrella review, nutrient, meta-analysis

## Abstract

Selenium is an essential trace metalloid element that is associated with fundamental importance to human health. Our umbrella review aimed to evaluate the quality of evidence, validity, and biases in the relationship between selenium intake and health-related outcomes according to published systematic reviews with pooled data and meta-analyses. Selenium intake is associated with a decreased risk of digestive system cancers, all-cause mortality, depression, and Keshan disease, when in children reduce the risk of Kashin-Beck disease. Additionally, selenium supplementation can improve sperm quality, polycystic ovary syndrome, autoimmune thyroid disease, cardiovascular disease, and infective outcomes. Selenium supplementation also has relationship with a decreased concentration of serum lipids including total cholesterol and very low-density lipoprotein cholesterol. However, no evidence has shown that selenium is associated with better outcomes among patients in intensive care units. Furthermore, selenium intake may be related with a higher risk of type 2 diabetes and non-melanoma skin cancers. Moreover, most of included studies are evaluated as low quality according to our evidence assessment. Based on our study findings and the limited advantages of selenium intake, it is not recommended to receive extra supplementary selenium for general populations, and selenium supplementation should not be continued in patients whose selenium-deficient status has been corrected.

## 1. Introduction

Selenium is an essential trace metalloid element that exists widely in several geographical sites, including the atmosphere, lithosphere, and hydrosphere of the Earth. The main forms of selenium in nature are inorganic compounds such as selenates, selenides, and selenates, and its chemical form can be affected by many environmental activities, such as the absorption and deposition of soil, burning of fossil fuels, and microbial biomethylation. It has been presented that selenium had potent antioxidant activity and was of fundamental importance to human health (1, 2). Its role as an essential mineral was first recognized in 1957 by scientists as having the advantage of preventing lesions in animal tissues (3). The different forms of selenium include selenomethionine, sodium selenite, and methylseleninic acid. Selenium can be obtained from selenium-rich foods, including nuts, seeds, beef, and fish (4). Generally, selenium in food exists in both organic and inorganic forms, such as selenomethionine, selenate and selenite, while the main selenium compounds in supplements are inorganic forms (2). The total amount of selenium in humans is approximately 20 mg and has shown great potential as an antioxidant for the maintenance of cellular homeostasis and metabolism. Previous studies reported that selenium might have associations with the improvement of dermatological conditions, immune system function and cancer preventing (5, 6). However, both excessive and deficient selenium concentrations in the body can result in a series of adverse health outcomes. For example, selenium deficiency can cause male infertility and Keshan disease, whereas selenium excess may induce functional failure in the liver or kidneys (7). Although the association between selenium intake and multiple health outcomes has been assessed in an increasing number of many systematic reviews and meta-analyses of diverse qualities, there is still a lack of literature comprehensively evaluating the overall connection between multiple health outcomes and selenium. Therefore, we aimed to provide an overview of the associations between selenium intake and health-related outcomes that had been detected in other meta-analyses to draw more convincing conclusions.

## 2. Literature research and methods

### 2.1. Literature research

This study was conducted using electronic databases (PubMed, Embase, Web of Science and Cochrane Database) from inception to April 2023. Systematic reviews and meta-analyses of interventional and observational articles were searched based on (selenium) and (scientific reviews * OR meta-analyses *). The 2020 Sign Guidance for scientific review and meta-analysis was also referenced in the study search (8, 9). Two investigators (PZW and YH) independently selected qualified articles after screening the titles, abstracts and reviewing the full-text. Any discrepancies in the selection process between the two investigators were addressed with the assistance of a third investigator (LRL).

### 2.2. Review method

Numerous systematic reviews and meta-analyses on various health-associated outcomes related to selenium intake were systematically searched and evaluated. Systematic reviews without pooled data were excluded from our study because daily selenium intake could be measured using a specific dose.

### 2.3. Eligibility criteria

Meta-analyses describing the correlation between selenium intake and various health outcomes were contained in our review regardless of the demographic characteristics of the respondents. This study did not cover other types of articles (cohort articles, randomized controlled trials [RCTs], and non-randomized controlled trials [NRCTs]), case–controlled articles, reviews, case reports, or letters) or meta-analyses written in languages other than English. When there were more health results within a meta-analysis, the data were extracted separately for the respective results. When different articles evaluated the same health results, we extracted data from the study with a larger number of participants. Articles reporting selenium intake in animals or laboratories were excluded. Moreover, studies that only assessed selenium concentration in the blood and other tissues were excluded because the internal selenium level could not reflect the dose of selenium supplements. Furthermore, due to the various concentration of blood selenium in participants among selenium-rich or selenium-poor areas, potential bias might exist when conducting selenium intake at same dosage in populations from different countries and districts.

### 2.4. Data extraction

Two authors (PZW and BC) acquired the following data in an independent manner from qualified meta-analyses: (1) publication year, (2) the first author’s name, (3) category of exposure (patterns of selenium intake), (4) healthy results, (5) number of included studies, (6) number of participants in each study, (7) study design (cross-sectional, case–control, cohort, RCTs, and NRCTs), (8) type of comparisons (highest versus lowest, any versus never and increment or reduction of any dose of selenium intake), (9) Cochran’ s Q test *p* value, (10) Egger’ s test *p* value, (11) the estimated summary effect (SMD, standard mean difference; WMD, weighted mean difference; OR, odds ratio; RR, relative risk) and corresponding 95% confidence intervals (CIs), (12) *I*^2^ statistic value, (13) effect model (random or fixed), and (14) follow-up time. The *p* value for nonlinearity was also extracted if a dose–response analysis was performed in a meta-analysis. If any discrepancy occurred during the extraction process, a third investigator (LRL) determined the final resolution.

### 2.5. Assessment of methodological quality of included studies and quality of evidence

AMSTAR, a strategy consisting of 11 items that has proven to be a reliable standard for assessing the quality of scientific reviews and meta-analyses, was utilized to evaluate the methodological quality of the covered articles (8). In addition, Grading of Recommendations, Assessment, Development, and Evaluation (GRADE) was used to assess the quality of evidence for the different health-related outcomes presented in our review. Evidence evaluation was classified into “high,” “moderate,” “low” and “very low” quality to making recommendations.

### 2.6. Data analysis

Data on exposure, each health outcome, and the estimated summary effect with 95% CI were extracted if available (10, 11). For one meta-analysis containing both case–control and cohort articles, the available data were extracted separately if possible. To estimate the heterogeneity between the covered articles, the *I*^2^ test and Cochran’s *Q* test were utilized, and Egger’s test was performed to calculate the publication bias in the respective studies (12). If available, the dose–response relationships in the meta-analyses are also presented. The standard (*p* < 0.05) was set for both the Egger’s test and heterogeneity.

## 3. Results

### 3.1. Characteristics of studies

**Figure 1:**
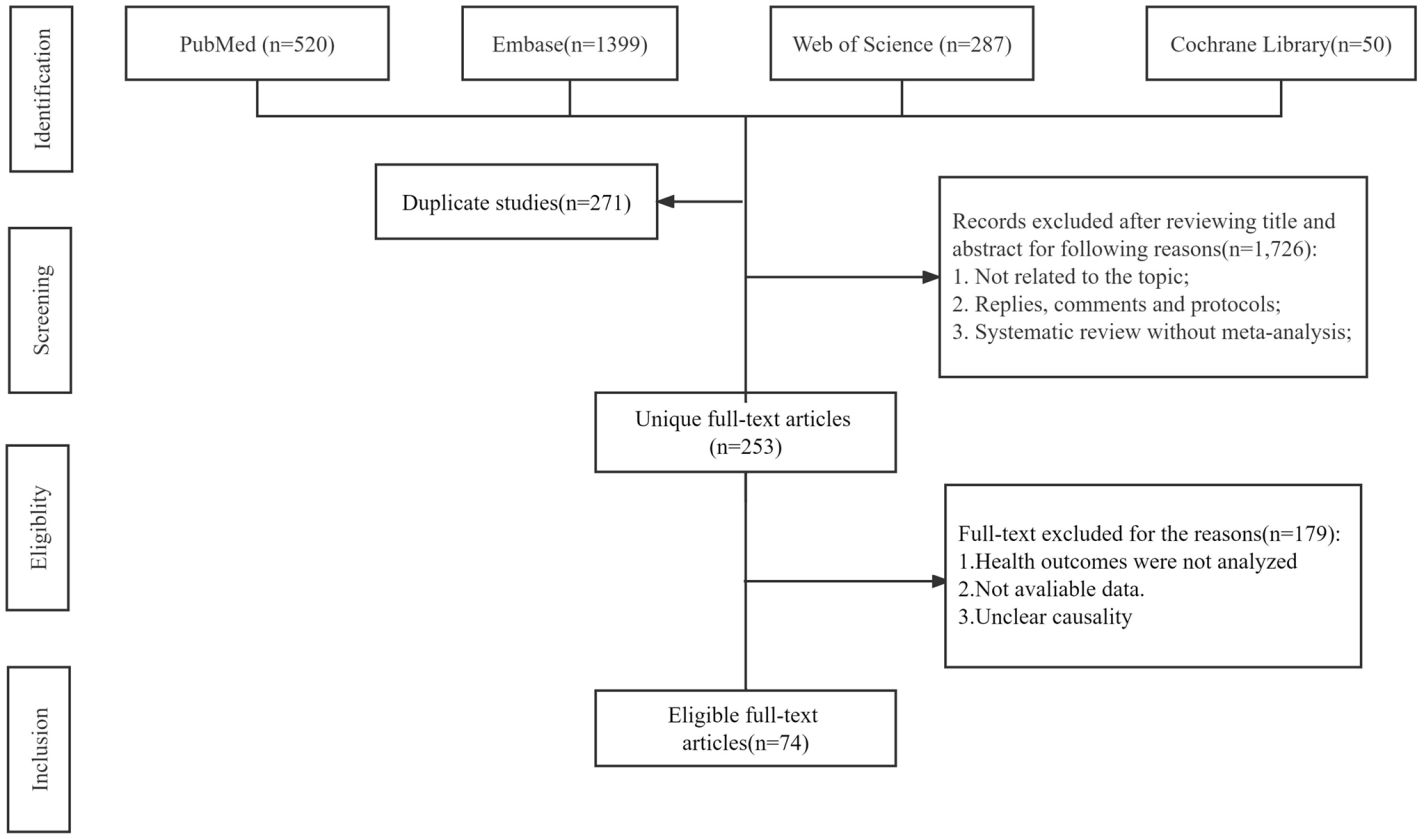
Flowchart of the systematic search and selection process.

**Figure 2:**
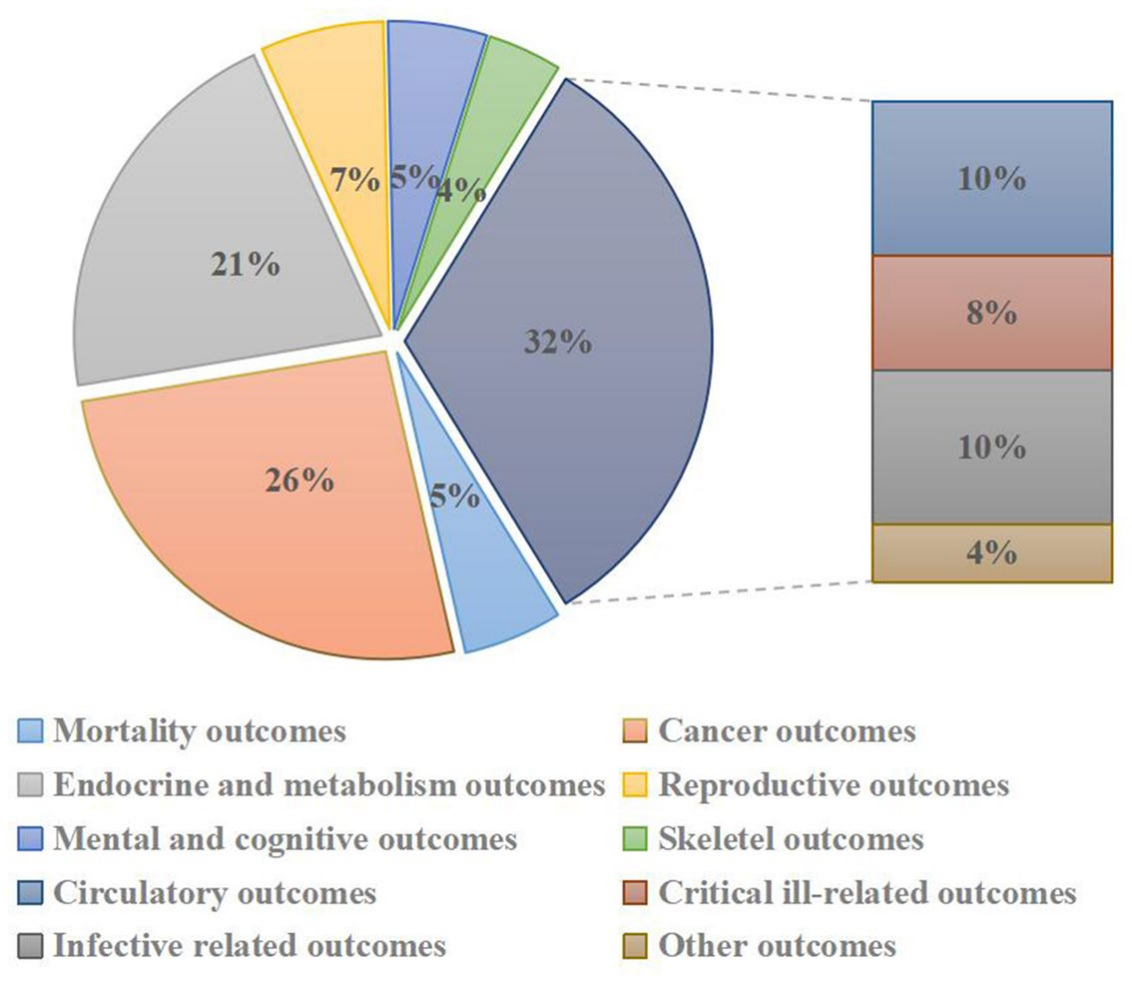
Pie chart of heath outcomes related to selenium intakes.

A flowchart of the systematic search and literature selection processes is shown in Figure 1. Our search identified 2,250 articles and eventually included 76 meta-analyses for this umbrella review; we retrieved 96 unique outcomes for both dietary and supplementary selenium intake (Figure 2). Characteristics of the association between selenium intake and multiple health-related outcomes are shown in Table 1 and Supplementary Tables 1, 2.

**Table 1:** Associations between selenium intakes and mortality and cancer outcomes.

Outcome	Author-Year	Type	Population	No. of cases/total	Metrics	Estimates	95%CI	No. of studies	Cohort	Case control	Cross-sectional	RCT	Effects model	*I*^2^	*Q* test p value	Egger test *p* value
Mortality outcomes																
*Significant associations*																
All-cause mortality	Jayedi, 2018	Diet	Adults	10,285/141,404	RR^a^	0.79	0.73, 0.85	3	3	0	0	0	Random	0	0.95	NA
*Insignificant associations*																
All-cause mortality	Bjelakovic, 2007	Supplement	Adults	NA/1,993	RR^a^	0.85	0.68, 1.07	3	0	0	0	3	Random	0	NA	NA
Cancer outcomes																
*Significant associations*																
All cancer	Lee, 2009	Supplement	Adults	NA/152,538	RR^a^	0.76	0.58, 0.99	8	0	0	0	8	Random	NA	NA	NA
All cancer	Kuria, 2020	Diet	Adults	NA/579,878	RR^b^	0.96	0.92, 0.99	106^c^	NA	NA	0	0	Random	30.6	0.003	NA
Gastrointestinal cancer	Bjelakovic, 2004	Supplement	Adults	164/6,077	RR^e^	0.49	0.36, 0.67	4	0	0	0	4	Random	0	0.94	NA
Liver cancer	Vinceti, 2018	Supplement	Adults	135/6,326	RR^a^	0.52	0.35, 0.79	4	0	0	0	4	Random	12	0.34	NA
Liver cancer	Kuria, 2020	Diet	Adults	NA/579,878	RR^e^	0.78	0.68, 0.90	14^c^	NA	NA	0	0	Random	11.2	0.331	NA
Pancreatic cancer	Wang, 2016	Supplement	Adults	1424/132,165	RR^a^	0.659	0.489, 0.889	6	3	3	0	0	Random	47.6	0.089	0.766
Pancreatic cancer	Chen, 2016	Diet	Adults	980/110,817	OR^a^	0.47	0.26, 0.85	6	3	3	0	0	Random	82.8	0.000	NA
Skin cancer	Kuria, 2020	Diet	Adults	NA/579,878	RR^b^	1.12	1.04, 1.20	21^c^	NA	NA	0	0	Random	0	0.970	NA
*Insignificant associations*																
Breast cancer	Vinceti, 2018	Supplement	Adults	94/2,260	RR^a^	1.44	0.96, 2.17	3	0	0	0	3	Random	0	0.78	NA
Breast cancer	Kuria, 2020	Diet	Adults	NA/579,878	RR^a^	1.89	0.69, 5.52	2^c^	NA	NA	0	0	Random	0	0.527	NA
Head and neck cancer	Vinceti, 2018	Supplement	Adults	22/2,811	RR^a^	1.22	0.52, 2.85	2	0	0	0	2	Random	0	0.79	NA
Colorectal cancer	Vinceti, 2018	Supplement	Adults	159/20,259	RR^a^	0.74	0.41, 1.33	3	0	0	0	3	Random	48	0.15	NA
Colorectal cancer	Kuria, 2020	Diet	Adults	NA/579,878	RR^e^	1.04	0.94, 1.16	14^c^	NA	NA	0	0	Random	0	0.562	NA
Esophageal cancer	Vinceti, 2018	Supplement	Adults	8/2,811	RR^a^	0.53	0.12, 2.28	2	0	0	0	2	Random	0	0.47	NA
Esophageal cancer	Hong, 2016	Diet	Adults	701/9262	RR^d^	1.01	0.99–1.03	4	1	3	0	0	Random	0	0.628	0.738
Melanoma	Vinceti, 2018	Supplement	Adults	32/3,277	RR^a^	1.28	0.63, 2.59	3	0	0	0	3	Random	0	0.98	NA
Non-melanoma skin cancer	Vinceti, 2018	Supplement	Adults	NA/5,661	RR^a^	1.23	0.73, 2.08	4	0	0	0	4	Random	58	0.07	NA
Lung cancer	Vinceti, 2018	Supplement	Adults	299/20,259	RR^a^	1.03	0.78, 1.37	3	0	0	0	3	Random	28	0.25	NA
Lung cancer	Kuria, 2020	Diet	Adults	NA/579,878	RR^e^	1.08	0.89, 1.31	4^e^	NA	NA	0	0	Random	25.7	0.257	NA
Bladder cancer	Vinceti, 2018	Supplement	Adults	146/20,259	RR^a^	1.10	0.79, 1.52	3	0	0	0	3	Random	0	0.73	NA
Bladder cancer	Kuria, 2020	Diet	Adults	NA/579,878	RR^abe^	1.16	0.76, 1.78	2^c^	NA	NA	NA	2	Random	0	0.817	NA
Prostate cancer	Sayehmiri, 2018	Supplement	Adults	23,994/45,638	RR^a^	0.9	0.74, 1.09	9	0	0	0	9	Random	54.3	NA	NA
Prostate cancer	Sayehmiri, 2018	Diet	Adults	5,206/80,020	RR^a^	1	0.98, 1.02	7	2	5	0	0	Random	0	NA	0.007

CI, confidence interval; NA, not available; OR, odds ratio; RCT, randomized controlled trial; RR, relative risk.

a Highest versus lowest.

b >55 μg/day versus never.

c The number of doses.

d 10 μg/day selenium intake increase.

e All dose versus never.

### 3.2. Mortality

Dose–response relationship revealed that the higher dose of dietary selenium intake (10-μg/day increment) than the lowest category might related to a lower risk of all-cause mortality in adults (RR: 0.79, 95% CI: 0.73, 0.85). In addition, every 0.2 μmol/L increment in circulating selenium concentration was associated with a decreased risk of all-cause mortality by 11% in a linear fashion (*p* = 0.4) (13). However, another meta-analysis demonstrated that the highest supplementary selenium intake might not correlate with the risk of all-cause mortality compared to the lowest intake (RR: 0.85, 95% CI: 0.68, 1.07) (14).

### 3.3. Cancer outcomes

The highest vs. Lowest dose of supplementary selenium intakes could decreased the risk of total categories of cancers in adults (RR: 0.76, 95% CI: 0.58, 0.99) (15). Another study revealed that dietary selenium intake (>55 μg/day) was associated with a lower risk of all types of cancer than no dietary selenium intake (RR: 0.96, 95% CI: 0.92, 0.99) (16). For a single category of cancer, supplementary selenium intake is related to a reduction in the risk of gastrointestinal, liver, and pancreatic cancers (17–19), and dietary selenium intake can reduce the incidence of liver, pancreatic, and skin cancers (16, 20). However, compared to the lowest selenium intake, supplementary and dietary selenium intake does not prevent several cancers, including breast cancer, head and neck cancer, colorectal cancer, melanoma or non-melanoma skin cancer, lung cancer, bladder cancer, and prostate cancer (16, 18, 21). Moreover, dose–response calculations detected that 10 μg/day selenium intake was not linearly associated with the incidence of esophageal cancer (RR: 1.01, 95% CI: 0.99, 1.03) (22).

### 3.4. Endocrine and metabolism outcomes

According to a meta-analysis of observational studies in Asian adults, the highest versus the lowest dietary selenium level was related to a 23% decrease in the risk of metabolic syndrome without a linear relationship according to a meta-analysis of observational studies in Asian adults (23). In addition, for total autoimmune thyroid disease (ATID) without classification, several studies observed that patients treated with combination of supplementary selenium and anti-thyroid drugs had a lower serum level of serum free triiodothyronine (FT3) and serum four triiodothyronine (FT4). Moreover, supplementary selenium could decrease the levels of anti-thyroid peroxidase antibody, particularly in patients who underwent levothyroxine (LT4) substitution. However, selenium supplementation has no significant advantage in reducing the levels of both thyroid-stimulating hormone (TSH) and anti-thyroglobulin antibody (TGAb) (24, 25). After categorizing different types of ATID, studies revealed that a dose of 200 μg selenium intake (selenomethionine) once per day was effective in reducing anti-thyroid peroxidase antibody (TPOAb) levels and improving well-being and/or mood in patients with Hashimoto’s thyroiditis compared to a placebo after 3 months (26). Furthermore, selenium supplementation plus the standard anti-thyroid drug methimazole (MMI) resulted in a significant decrease in FT3 and FT4 levels (3 and 6 months) and an increase in TSH levels (6 and 9 months) among patients with Graves’ disease when compared to control participants (27). Moreover, meta-analyses have indicated that supplementary selenium intake could also prominently decrease glycemic indices, including the homeostasis model of assessment-estimated-cell function (HOMA-B) and homeostasis model of assessment-estimated insulin resistance (HOMA-IR), and increase the quantitative insulin sensitivity check index (QUICKI) (28, 29). However, a dose–response analysis found that above 60 μg of daily dietary selenium intake was associated with a higher risk of type 2 diabetes (30), and supplementary 200 μg/day of selenium intake could also have a linear relationship with diabetes incidence in Western participants (31). Moreover, no significant association was detected between selenium consumption and some glycemic indices such as insulin level, fasting plasma glucose, and hemoglobin A1c (HbA1c) (32).

### 3.5. Reproductive outcomes

Supplementary selenium intake may be associated with a significant improvement in male infertility. Selenium intake could increase the sperm morphology and motility compared with patients without supplementary selenium consumption (33). In addition, a meta-analysis involving only RCTs revealed that infertile patients with selenium supplementation of 100 or 200 g/day had apparent increases in sperm concentration and total volume (34). However, the same study reported no significant relationship between selenium supplementation and the total pregnancy rate compared to the placebo group.

A meta-analysis of 389 participants revealed a correlation between supplementary selenium intake and polycystic ovary syndrome (PCOS) in female. Daily dose of 200 μg selenium could increase the level of sex hormone binding globulin and total antioxidant capacity (TAC) with no significant heterogeneity (35). Moreover, regular supplementary selenium intake significantly decreased serum levels of total testosterone and cholesterol (36). These results suggest that selenium supplementation might mitigate health risks in patients with PCOS by alleviating oxidative stress and abnormal lipid metabolism, which has been proven to be a pathogenic mechanism of infertility and abnormal menstruation in patients with PCOS (37, 38).

### 3.6. Circulatory outcomes

In terms of supplementary intake in adults, a combination of cardiovascular drugs and selenium compared with placebo might be associated with a decreased risk of coronary heart disease by decreasing the serum levels of total cholesterol (TC, WMD: -2.11, 95% CI: −4.09, −0.13) and very low-density lipoprotein cholesterol (WMD: -1.35, 95% CI: −2.33, −0.37). and the results were not affected by the intervention of both sexes and daily dose (≤200 μg). Daily supplementary selenium intake also significantly increased systolic blood pressure (SBP, SMD: 2.02, 95% CI: 0.50, 3.55), but had no obvious influence on diastolic blood pressure (DBP) (39). Besides, a meta-analysis involved nearly two million participants demonstrated that supplementary selenium intake was evidently associated with a significant reduction in Keshan disease incidence (RR: 0.14, 95% CI: 0.12, 0.16) (40). However, regarding lipid metabolism in the general population, there is no apparent evidence that selenium supplementation has advantages or disadvantages in decreasing total triglyceride, low-density lipoprotein cholesterol, high-density lipoprotein cholesterol, and body mass index (39).

### 3.7. Skeletal outcomes

For children specifically, any utilization of supplementary selenium compared with placebo or not might be associated with a significant lower risk of Kashin-Beck disease, an endemic osteoarthropathy with ambiguous etiology, by approximately 87% (RR: 1.88, 95% CI: 1.51, 2.33) with no heterogeneity identification (41). Furthermore, pediatric patients with Kashin-Beck disease also benefit from regular selenium supplementation, which could improve radiographic structures (OR: 0.13, 95% CI: 0.04, 0.47) (42).

### 3.8. Outcomes in intensive care units

Several studies have evaluated the effectiveness of daily oral or intravenous selenium supplementation in adult patients in intensive care units (ICUs). Selenium supplementation did not improve or decrease the total mortality, risk of new infectious complications, length of hospital and ICU stay, new renal dysfunction events, overall survival (28-days, 3 months, and 6 months), or ventilator days (43, 44).

### 3.9. Infective outcomes

For clinical outcomes in patients with sepsis syndrome, studies found that selenium supplementation treatment was correlated to a reduced duration of vasopressor therapy time (SMD: −0.75, 95% CI: −1.37, −0.13), shorter length of ICU (SMD: −0.15, 95% CI: −0.25, −0.04) and hospital stay (SMD: −1.22, 95% CI: −2.44, −0.01) (45), and respiratory tract infections (OR: 0.624, 95% CI: 0.696, 0.545) (46). Another meta-analysis detected that supplementary selenium with a daily dosage of 200 μg/d in patients with metabolic diseases could clearly connect to a decrease in high-sensitivity C-reactive protein (hs-CRP, SMD: −0.44, 95% CI: −0.67, −0.21), which indicates a reduced grade of inflammation in the body (47). Although the same study also reported an increased serum level of CRP in patients who received regular daily selenium supplementation, the author considered this result unreliable because of the discrepancy in the forest plot.

Moreover, there was no significant association between selenium intake and several outcomes including total mortality (28-days, 3 months, and 6 months), new renal dysfunction events, or secondary infection events (45).

### 3.10. Other outcomes

There were significant associations between the selenium supplementations and preeclampsia in pregnant women that selenium yeast with a daily dose of 60 to 100 micrograms for a period from the first trimester until delivery might decrease the incidence of preeclampsia (RR: 0.28, 95% CI: 0.09, 0.84) (48). In addition, adults receiving both inorganic and organic supplementary selenium might not have significantly improved immune function and reduced infectious disease susceptibility because selenium did not have an apparent effect on the serum levels of any type of antibody or immunocyte (49).

### 3.11. Heterogeneity of included studies

Among the included 76 studies, 17 meta-analyses including 32 health outcomes reported a *Q* test *p* value of <0.10. Twenty-one meta-analyses including 44 health outcomes, showed a low degree of heterogeneity, with *I*^2^ < 25%. Eighteen and 11 meta-analyses, containing 28 and 18 unique outcomes, respectively, had moderate and high heterogeneity, which containing *I*^2^ ranging from 25 to 75 and > 75%. However, eight studies including 10 health outcomes, did not report heterogeneity and therefore could not be reanalyzed.

### 3.12. Publication bias of included studies

Seven meta-analyses did not report significant publication bias according to the presented Egger’s test *p* value, and 12 meta-analyses containing 25 unique outcomes detected a significant publication bias. Moreover, a total of 26 studies including 38 health outcomes, did not perform or mention the Egger’s test for publication bias.

### 3.13. AMSTAR and GRADE evaluation of included studies

The results of the AMSTAR and GRADE evaluations are shown in Supplementary Tables 3–6. Supplementary Tables 3, 4 presents detailed information on the AMSTAR evaluation; the mean AMSTAR score was 8.03 (range, 6–10; interquartile range [IQR]: 8–9). This is because most of the included articles did not display information on the excluded studies, which is one of the critical domains. In terms of GRADE categorizations, the vast majority (90.79%) of included 76 articles were rated as “very low” and “low” when 9.21% of articles were rated as “Moderate,” respectively. One reason for the low evidence of strength is the existing bias in the studies. Moreover, most of the studies involved did not reach the width, breadth, or magnitude of the bonus items. The deficiency of the dose–response gradient was also a significant factor affecting the low-rate evaluation.

## 4. Discussion

### 4.1. Principal findings and interpretation

In summary, 76 meta-analyses involving 96 unique health outcomes related to dietary and supplementary selenium intakes were identified in this umbrella review. Regarding mortality and cancer outcomes, dietary selenium intake at the highest dose could decrease the risk of all-cause mortality in adults compared to the lowest dose. Dietary selenium is also associated with a decreased incidence of all types of carcinomas, particularly liver, pancreatic, and skin cancers. In addition, any dose of supplementary selenium might be related to a lower risk for overall categories of tumors, especially gastrointestinal, liver, and pancreatic malignancies. For other non-cancer health outcomes, dietary selenium consumption may be correlated with a decreased risk of metabolic syndrome and total depression, whereas selenium supplementation may reduce the incidence of postpartum depression, Kashin-Beck disease, Keshan disease, and pregnancy preeclampsia. In addition, supplementary selenium combined with specific drugs could improve the symptoms and some disease-related indices of several conditions, including total ATID, Graves’ disease, depression, PCOS, and cardiovascular disease (CVD), compared to placebo. Supplementary selenium consumption ≤200 μg per day in adults was significantly linked with a better mood in patients with Hashimoto’s thyroiditis. Moreover, infertile males with a daily dose of ≤200 μg selenium supplementation were observed to have better sperm qualities in sperm motility and morphology. Furthermore, supplementary selenium intake may downregulate some inflammatory markers, decrease blood lipid levels, and regulate SBP in the general population. However, supplementary selenium might not improve the physical condition of patients in ICUs, and it cannot control the immune ability of users. Moreover, both dietary and supplementary were associated with an increased incidence of type 2 diabetes without effecting several glycemic indices including insulin level, fasting plasma glucose, and hemoglobin A1c.

A large amount of selenium in the human body exists in selenomethionine, selenocysteine, proteins containing these amino acids, and other organic forms (50, 51). Selenium concentration varies in organs, from up to approximately 45% of the total selenium content in skeletal muscles to only approximately 4% in the kidneys (52). The average concentration of selenium in serum fluctuates at 60–120 μg/L, which has been commonly used as a standard clinical indicator of selenium status to evaluate excess or deficiency (53). Other indicators, including glutathione peroxidase activity and selenoprotein levels, can indirectly reflect the estimated selenium content in the body. In general, deficiency symptoms can be observed in patients with plasma selenium levels <85 ng/mL (54). The average plasma selenium concentration differs depending on geographical area and is relatively high in North America, with serum selenium levels found to be lower in Asian and European countries (55, 56), which is mainly effected by selenium accumulation in environment. Geological research presents that the middle part of China belongs to Se-poor belt, in which the mean soil selenium concentrations are approximately 0.13 mg/kg. Thus, populations living in the Se-poor belt in Central China are more possible to get Kashin-Beck disease and Keshan disease (57).

The main approach for selenium intake and accumulation is the ingestion of multiple products of plant and animal origin. The forms of selenium in animal products are the main organic compounds (mostly selenocysteine) that are easily consumed by humans. In contrast, the selenium accumulated in plants is an inorganic compound, including selenates and selenite, which can be converted into selenomethionine and selenocysteine by biochemical processes. In addition, the amount of dietary selenium differs and depends on the environmental location of living propagation (1, 58). Some protein-rich foods such as seafood, meat, fish, milk, cereals, and several kinds of plants, including mushrooms and garlic, have been detected to contain higher amounts of selenium than other foods (1, 59, 60). Protein-poor foods, such as fruits and vegetables, have low levels of selenium because they primarily occur in the protein fraction (61). Cereal products account for half of the daily intake of selenium, whereas animal foods account for approximately 35% (1). Absorption of selenium by the human body is influenced by multiple factors. Age, physiological condition, selenium amount, bioavailability of selenium compounds (62), several diseases, including consistent diarrhea or nutrient deficiencies, and even genetic polymorphisms (63) might affect the process of selenium absorption. For example, stable human isotope studies have indicated that some forms of selenium, including ingested selenomethionine and selenate, exhibit higher gut absorption than other forms of organic or inorganic selenium (64). Hence, it is noteworthy that there is a disparity between the amount of selenium intake and absorption. Selenium is absorbed in the small intestine following ingestion. After crossing the intestinal brush border membrane, inorganic selenium absorption depends on the sodium-facilitated and energy-dependent system utilized by sulfate, whereas organic forms of selenium are mediated through the same active sodium-dependent transport system as their sulfur-containing counterpart, methionine (65, 66). A study involved Norwegian women found that daily dosage of 100 μg extra dietary selenium intake for 6 weeks could increase the serum level of selenium from 115 μg/L to 135 μg/L, while 200 μg extra selenium intake from food consumption could result in an increased serum level of selenium from 122 to 159 μg/L (67). Another study comparing selenium kinetics showed that the overall amount of selenium absorbed increased with supplementation and the selenium concentration in whole blood continued to increase for several months after the end of supplementation (68). Thus, dietary and supplementary selenium intake could be utilized as noninvasive biomarkers to evaluate the status of selenium and explore its impact on health outcomes.

**Figure 3:**
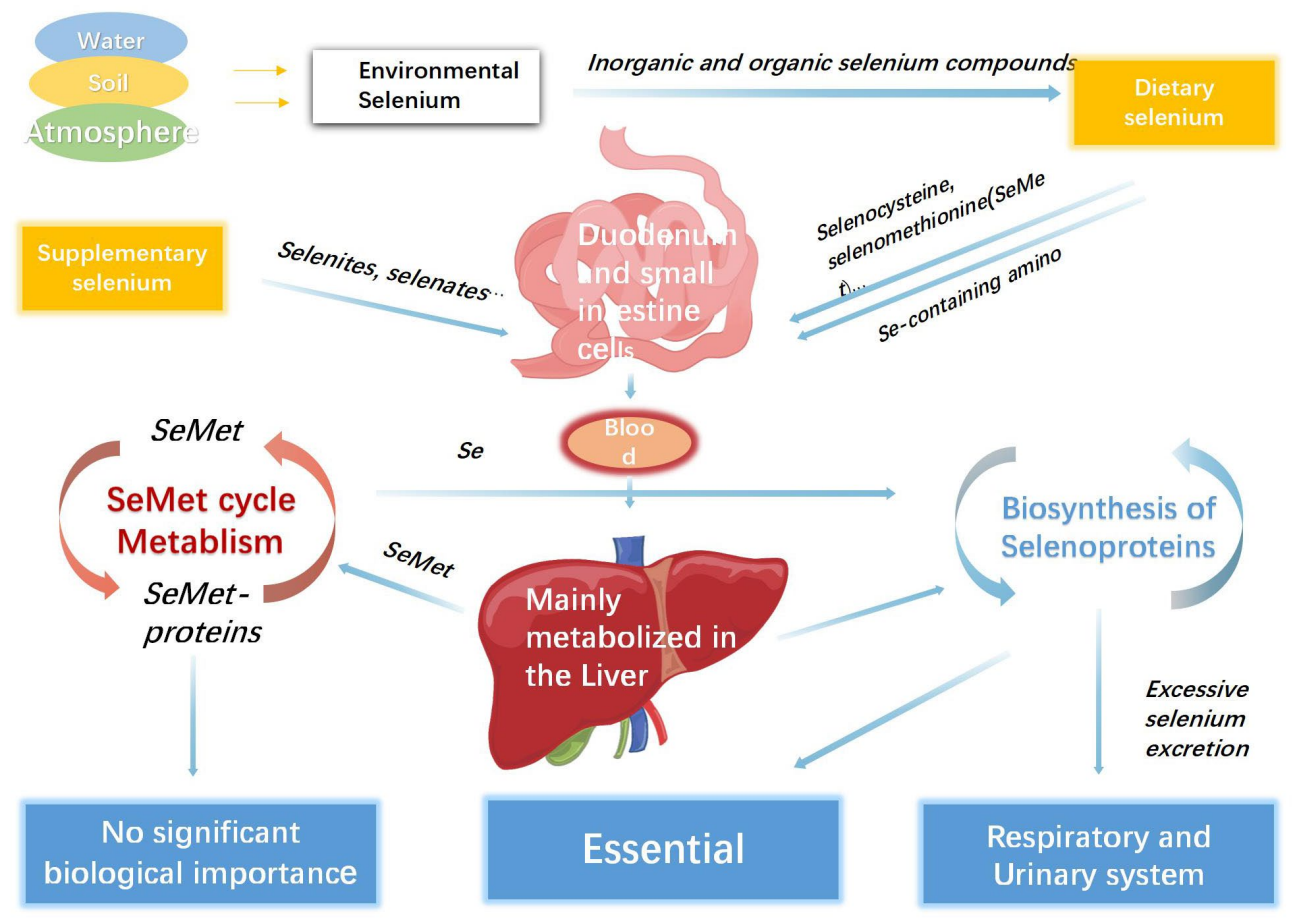
The process of selenium metabolism in human body.

Selenium deficiency is prevalent worldwide, which is mainly related to low concentrations of environmental selenium (1), and approximately one billion people worldwide lack sufficient selenium in their diet (69). However, it is difficult to define the specific threshold of selenium deficiency because of the discrepancies between sex, age, and geographical districts. Selenium overload or deficiency can cause substantial disease loads in both developed and developing countries. One cross-sectional comparative study found that after analyzing blood samples using an inductively coupled plasma-optical emission spectrometry system, pregnant women had significantly lower selenium levels than the control group, which might possibly result in an increased risk for pregnancy-associated complications (70). For the wide variation of selenium deficiency across countries and agencies, the recommended dietary allowances need to fluctuate within an appropriate range. Thus, it is difficult to determine a specific dose for daily selenium intake regardless of nutrition or disease status. Galan-Chilet and his team conducted a gene–environment interaction population-based study and eventually suggested that the optimal daily intake of selenium was 55 μg for general population (71), while another study reported that the recommended daily dose for children was 25 μg (53). Moreover, for infants, including neonates, 2 μg/kg per day of selenium is sufficient to meet the requirements of growth and development (72, 73). It is generally believed that the estimated harmless daily dose of selenium intake is less than 800 μg, whereas the dose that causes adverse toxic effect is approximately 1,600 μg per day (56, 59). In addition, based on the latest opinion from the EFSA Panel on Nutrition, Novel Foods and Food Allergens the tolerable upper intake level for selenium is 255 μg/day for adults (including pregnant and lactating women) (74). As mentioned above, both proper dietary and supplementary selenium would increase the concentration of serum selenium and improve symptoms caused by mild selenium deficiency in patients. However, for patients who are diagnosed with moderate or severe selenium deficiency and accept continuous selenium supplementation, regular laboratory tests for serum selenium are indispensable because of the toxicity induced by excessive organic or inorganic selenium (4, 75). Serum selenium is thought to be the most important indicator for maintaining homeostasis; however, most effects of selenium on metabolism are attributed to its incorporation into a family of proteins called se-containing proteins (selenoproteins) (76). The difference between selenium and other micronutrients is that it is directly involved in the composition of proteins in the form of selenocysteine (Sec) (64, 77). Twenty-five members of the selenoprotein family, which have diverse biological functions and tissue distributions (78). Most members function as enzymes and factors that regulate redox reactions and immune responses, particularly lipid membranes, muscle metabolism, and incremental processes (79, 80). Representative selenoproteins include glutathione peroxidases (GPXs), iodothyronine deiodinases (DIOs), selenophosphate synthetases, transporters, and transmembrane proteins (76). After absorption in the duodenum and small intestine, which seemed to be irrelevant to the internal selenium concentration, the consumed selenium-containing compounds were transferred into the blood across intestinal epithelial cells. Most of these molecules bind to glutathione with the formation of disulfides, which are eventually transported into the liver for further metabolism (64, 81, 82). Selenium in the liver has two major consumption processes: entry into the selenoprotein synthesis pathway and transformation into excretory metabolites (83). Plant-derived selenomethionine (SeMet) enters circulation without changing SeMet protein synthesis. SeMet can also release a portion of dissociative Se^0^ to participate in the selenoprotein cycle that develops Sec-containing selenoproteins, which are the most essential selenoproteins in the human body. Selenoprotein P (SELENOP), is synthesized in the liver and secreted into the serum for selenium delivery to the peripheral tissues (84, 85). Excess selenium compounds are excreted from the urinary and respiratory tracts in the form of trimethylselenonium and dimethyl selenide (86, 87). In addition, the urinary metabolites of superfluous selenium exist in the selenosugar branch (88, 89). However, the involvement of selenosugars in selenium recirculation remains controversial (Figure 3).

The biological importance of selenium is attributed to its occurrence in three types of selenoproteins produced by human cells: SeMet-containing proteins, selenoproteins, and Se-binding proteins (76, 90, 91). SeMet is synthesized in some plants and fungi and enters the human body through food. After recognition by the methionine-specific aminoacyl-tRNA synthetase, SeMet participates in protein biosynthesis. However, sufficient evidence has shown that SeMet-containing proteins do not cause obvious biological effects, except for selenium reservation (91). However, the specific roles of these Se-binding proteins in metabolism remain unclear. Since there is no free Sec in the body, selenoproteins are extremely important in human and animal bodies, and their biosynthesis involves several complex stages with the participation of selenocysteinyl tRNA^SeRSeC^ (92). In eukaryotes, tRNA^SeRSeC^ is acylated by serine and transformed into Ser-tRNA in the presence of adenosine triphosphate (ATP), which is then induced by seryl-tRNA synthetase (92). Subsequently, O-phosphoseryl-tRNA[Ser]Sec kinase (PSTK) phosphorylates the hydroxyl moiety of serine in an ATP-dependent manner to produce phosphoseryl-tRNA^SeRSeC^ (93). With the involvement of selenophosphate synthetase 2(SEPHS2), Sec-tRNA synthase (SEPSECS) catalyzes a reaction in which phosphorylated serine is substituted by a selenium atom, resulting in Sec-tRNA^SeRSeC^, which decodes the UGA codon as selenocysteine instead of a stop codon and finally forms a Sec-selenoprotein (7, 94, 95). In addition, the encoding of UGA for selenocysteine incorporation requires the existence of a unique mRNA element named the selenocysteine insertion sequence (SECIS), which has diverse locations in prokaryotes and eukaryotes (96). Moreover, some protein factors, including nucleolin, ribosomal proteins, sec-specific eukaryotic elongation factors, and SECIS-binding protein 2(SBP2), are also crucial for the biosynthesis of selenoproteins (91, 97).

Reactive oxygen species (ROS), which are derived from the aerobic respiration of cells and a number of exogenous factors, control diverse aspects of physiological processes and result in oxidative stress to eventually damage proteins, DNA, and lipids irreversibly (98–101). Numerous studies have shown that the oxidative stress response can promote the development of inflammation and thus has significant relationships with occurence and development of multiple diseases (102–106). Antioxidant properties are the most prominent characteristics of many selenoproteins, including the GPXs family, selenoprotein T (SELENOT), and selenoprotein W (SELENOW) (107). Selenoproteins play pivotal antioxidant roles by regulating glutathione and thioredoxin, which are believed to be the principal antioxidant systems in humans (108, 109). Glutathione, a tripeptide consisted by γ-l-glutamyl-l-cysteinyl-glycine, can eliminate the destructive effects of hydrogen peroxide and other peroxides (110, 111) to prevent red blood cells, cell membranes, and hemoglobin from oxidation (112). GPXs promote the biosynthesis of glutathione and increase the amount of glutathione to protect DNA against oxidative damage. Another Se-containing enzyme, glutathione reductase, catalyzes the oxidation of glutathione to its reduced form to maintain a sufficient concentration of reduced glutathione (1, 113). The thioredoxin system is an important antioxidant system comprising thioredoxin reductase, thioredoxin, and nicotinamide adenine dinucleotide phosphate (NADPH) (114, 115). Thioredoxin reductase is a selenoprotein, wherein Sec is located at the penultimate position of the polypeptide chain (116). Three thioredoxin reductases are localized in the cytoplasm, nucleus, and mitochondria, providing reducing equivalents to disulfide bonds (117). Disulfide bonds, which are the basis for the redox properties of selenopeptides, play a crucial role in maintaining the stability of protein structures. Research has shown that Sec can be easily oxidized to its corresponding diselenide form (118, 119), which has proven to be an effective method for establishing Se-Se bridges, which substantially belong to disulfide bonds (77). SELENOP is involved in the storage, transport, and supply of selenium and can also bind heavy metal atoms and play a significant role in defense against oxidative stress, which might benefit from several Sec residues in its amino acid sequence (91). Owing to the function of selenium transport to peripheral blood tissues, the SELENOP serum concentration is considered an effective biomarker for selenium status and overall body condition estimation (120).

The association between selenium intake and health outcomes may be explained by its effects on ferroptosis. The concept of ferroptosis, defined as “a non-apoptotic form of cell death that is dependent on iron and caused by lipid peroxidation,” originates from precision oncology (121). Ferroptotic cell death may promote tumor cell development by increasing the inflammatory response. In addition, tumor tissues avoid ferroptosis through metabolic reprogramming, lactate, and master growth regulators, which decrease iron utilization and ultimately result in a high level of iron that supports tumor cell proliferation and participates in the synthesis of metabolic enzymes in tumor cells (122, 123). Studies have proved that ferroptosis plays a vital role in several cancers, neurological disorders, and inflammatory diseases (124–127). Ferroptosis is primarily caused by the inactivation of GPX4, which is recognized as the central enzyme that limits lipid peroxidation; thus, selenium supplementation increases the synthesis of GPX4 to prevent ferroptosis (128). Selenium also activates transcription factors TFAP2c and Sp1 to further enhance the activity of GPX4 and protects neurocytes (129).

Cellular selenium also play crucial roles in cell cycle progression and apoptosis. Apoptosis is a physiological form of cell death that is associated with various diseases, including malignancies (130). Cytometric analysis suggested that supplementation with selenium compounds accelerated cell cycle progression, and Se-deficient cells showed higher apoptosis than the experimental group (131). Selenium deprivation induces cell cycle arrest and apoptosis by activating the caspase cascade, which is considered a possible mechanism to decrease the incidence of tumors (132). Several studies have revealed that selenium intake can suppress tumor development by affecting the expression of regulatory genes related to cell proliferation and apoptosis (131). However, some malignancies, including hepatocellular carcinoma and colon cancer, can tolerate selenium deficiency and avoid its anti-cancer properties (133, 134). Similarly, both insufficient and supranutritional dosages of selenium supplementation may impair spermatogenesis by regulating *PI3K/AKT-*mediated apoptosis of testicular cells (135) and weakening the antioxidant capacity of sperm (136). Selenium deficiency in women also induces necroptosis of uterine smooth muscle cells through *ROS/MAPK* signaling pathway, which may be a potential mechanism for lower pregnancy rates (137). In contrast, clinical trials concentrating on male infertility demonstrated that moderate selenium therapy prominently reduced cell apoptosis and DNA fragmentation in sperm, and eventually enhanced semen parameters (138, 139).

Previous studies have presented that selenium intake also affects the status of the human immune system. When selenium is diminished, susceptibility to infections and cancers increases because it impairs innate and adaptive immunity (140, 141). A study undertaken in Finland showed that Se-deficient individuals who accepted daily dietary selenium for 200 μg had higher antibody concentrations than the control group, and another double-blind trial detected a dose-dependent increase in T-cell proliferation after selenium supplementing (142, 143). After receiving selenium supplementation, the immune functions of neutrophils and lymphocytes significantly increased to produce more cytokines (143, 144). Experiments based on humans have proved that selenium and viral infections had significant interactions (145–147). The vast majority of most notorious pandemic viruses, including HIV, COVID-19 and Zika, have RNA genomes, which enable them to adapt diverse environment rapidly through several unique characteristics. First, small genome size allow them to self-replicate fastly. Then, RNA viruses have magnitude higher mutation rate than DNA viruses, enhancing their ability to continuously evade immune surveillance (148, 149). RNA viruses can inhibit DNA synthesis by impairing the function of thioredoxin reductase (TR), a selenoprotein in mammals. Because of the antisense complementarity between RNA virus mRNAs and host mRNAs encoding isoforms of thioredoxin reductase, the synthesis of the targeted isoform of TR can be interfered, which leads to a low level DNA synthesis and eventually results in an increased RNA synthesis. Thus, selenium deficiency decreases TR concentration to effect DNA synthesis and promote RNA replication of viruses. Conversely, replete selenium status may inhibit the RNA viral replication by creating more favorable conditions for DNA synthesis (150). There are also some studies considering that the incidence and progression of corona virus disease-19 (COVID-19) might be related to dietary selenium status. Many viral infections, including COVID-19, are associated with a heightened level of oxidative stress and inflammation. COVID-19 infection causes a decreased expression of host selenoproteins and subsequently impairs the ability of redox regulation. The latest research reported that the main protease of COVID-19 could target several selenoproteins, including TR1, γ-glutamate cysteine ligase (GCLC) and SELENOP, promote proteolytic degradation and eventually disrupt the Thioredoxin and Glutaredoxin Redox Cycles, the necessary process of DNA production (151). Selenium supplementation may restore antioxidant capability by enhancing the synthesis of selenoproteins, and promote the proliferation of lymphocyte cells (145, 152, 153). This explanation can also be a convictive evidence to support our result that selenium had advantage in preventing the incidence of Keshan disease. Keshan disease could be categoried into viral myocarditis, which attributed to virus invasion secondary to selenium deficiency. Recent studies have suggested that coxsackie virus B3(CVB3), a enterovirus with single-stranded RNA molecule, might be a contributing factor in the pathogenesis of Keshan disease (154–156). Under the status of selenium deficiency, benign CVB3/0 is more possible to get virulent mutatation and ultimately contribute towards the development of myocarditis (157). The mechanisms that explain the strengthening effect of selenium on immunity are as follows: stabilizing ROS that play vital roles in immune cell signaling; affecting oxidative burst in both phagocytic and non-phagocytic cells; increasing IL-2 and IL-2 receptors; regulating the differentiation of macrophages; and promoting the combination of nuclear factor-kappa B (NF-κB) and target gene regions in Jurkat T cells (158–161). Increased immunity may have positive effects on carcinogenesis and cancer progression, such as lymphomas and hepatocellular carcinoma (162, 163). However, according to our research, the possible protective effect of selenium intake against cancer is not applicable for most cancers. Although the highest versus lowest selenium intake may reduce the risk of gastrointestinal and pancreatic cancers, no significant associations between selenium intake and breast, colorectal, lung, and esophageal cancers have been demonstrated (16, 18). More interestingly, the same study also revealed that non-melanoma skin tumors were an exception, in that dietary selenium intake might increase the risk of skin cancers without melanomas by 12% (RR: 1.12, 95% CI: 1.04, 1.20). However, because insufficient primary studies were included based on our search strategy, the association between selenium intake and immunity is still controversial.

Another hypothesis presents a possible antitumor mechanism of selenium intake due to its diverse distribution in different organs. For example, the liver is richly supplied with selenium because of its indispensable role in selenium metabolism, and research has found that both dietary and supplementary selenium protect the liver against deadly hepatocellular carcinoma (16, 18), which might justify the rationality of this mechanism. However, there is little comprehensive evidence on the association between selenium-containing organs, including the testis and kidneys, and cancer outcomes. From a genetic perspective, the results of a wide-angled Mendelian randomization analysis also questioned the cancer-preventing function of selenium. This study used single nucleotide polymorphisms associated with selenium levels from genome-wide analyses as instrumental variables and observed that genetically predicted selenium levels had no relationship with the risk of multiple specific cancers (164).

Apart from strengthening immunity multidimensionally, a meta-analysis of over 140,000 participants with 10,285 cases also attached importance to the fact that people with 10 μg/d dietary selenium intake would insignificantly decrease the risk of all-cause mortality compared with the lowest category (RR: 0.79, 95% CI: 0.73, 0.85) (13). Furthermore, subgroup analysis based on age suggested that decreased selenium concentration had a strong relationship with increased age, which might explain the potential mechanism of a higher incidence of a proinflammatory state in the internal environment of elderly people (165, 166). However, the author also indicated that because most of the included primary studies were based in Western countries, it would be prudent to generalize the conclusion to Asian populations.

In metabolism-related diseases, selenium disequilibrium is thought to be involved in the progression of multiple cardiovascular conditions. As described previously, selenium deficiency dysregulates the synthesis of selenoproteins to trigger the production of ROS, and then impairs cellular growth and remodeling of cardiomyocytes by inducing autophagy and apoptosis and affecting the redox-methylation balance (167). Trials on cultured human cardiomyocytes have shown that selenium insufficiency is associated with decreased expression of proteins that regulate respiratory oxidative phosphorylation (168). Selenium deficiency also greatly elevates NO levels and increases protein S-nitrosylation. A recent study in animal models found that the myocardial tissue of pigs fed a selenium-deficient diet for more than 2 weeks selenium-deficient diet feeding clearly presented adipose infiltration and fibrosis (168). The same study also detected that more proinflammatory molecules, such as interferon β and interleukin-1β were produced in the selenium-deficient group because multiple genes involved their upstream pathways, including *UPS18*, *DDX58*, *cGAS*, and *TLR3* were upregulated, which further supported the conclusion that selenium deficiency could resulted in mitochondrial DNA damage and disorder energy production of cardiac cells (169). Moreover, several studies have reported that selenium supplementation may have cooperative effects in enhancing the efficacy of anti-thyroid drugs, and selenium intake at the highest dose could simultaneously improve the quality of life among autoimmune thyroiditis patients (170, 171). Interestingly, however, although the type 2 diabetes development might also be correlated to similar pathomechanisms containing inflammation, mitochondrial dysfunction and redox disturbance, a meta-analysis of both nonexperimental and experimental studies revealed that selenium intake had an increased risk of diabetes by 11% (RR: 1.11, 95% CI: 1.01, 1.22) (31, 172). Other adverse reactions should also raise awareness of selenium supplementation. As mentioned above, selenium intake might have a protective effect against several cancers, but excessive selenium consumption could increase genetic instability and ultimately induce carcinogenicity of lymphocytes and osteocytes (173). In contrast, other symptoms such as garlicky breath, hair or fingernail loss, allergic reactions, and dermatitis can also be caused by selenium supplementation (174, 175). According to a review by Hadrup (176), a human dose of 10,000 μg/kg can be associated with the risk of acute mortality, and the associated symptoms include vomiting, diarrhea, and respiratory failure. Therefore, caution should be exercised when supplementing the general population with selenium.

Of note, our study found that selenium intake might be associated with a significant lower risk of Kashin-Beck disease. However, although selenium deficiency has been proved as a main risk factor of this disease, recent evidence detected that its etiology could contain multiple factors, including inadequate intake and proportional disequilibrium of non-selenium nutrients and differentially gene expression (177). Thus, it is inappropriate to prevent Kashin-Beck disease through dietary or supplementary selenium intake only.

### 4.2. Strengths and limitations

This umbrella review provides the latest comprehensive overview of published studies on the relationship between multiple health outcomes and selenium intake. Standard methods, including AMSTAR and GRADE, were used to assess the methodological quality and strength of the included studies. In addition, many studies were meta-analyses of RCTs, which are recognized to have a higher quality than observational studies in terms of the grade of evidence. However, we acknowledge several potential limitations of this umbrella review. Firstly, To begin with, a large number of involved meta-analyses were considered as “low” and “very low” in GRADE categorizations, which was mainly because that most of studies did not offer the list of excluded articles. Similarly, insufficient participant numbers and plausible confounding factors also contributed to the imprecise results. Second, based on our selection criteria, we included only published meta-analyses; recent or unpublished studies may have been omitted. Third, a potential publication bias was inevitable because several eligible meta-analyses involved only a small number of studies and populations. Fourth, both the period of receiving selenium and the dosage were diverse in most meat analyses, which might have weakened the consistency of our findings to a large extent. In addition, we were unable to summarize the dose–response relationship between selenium intake and multiple health-related outcomes owing to the lack of primary data. Nevertheless, many meta-analyses included generally healthy participants, which may have minimized possible bias.

## 5. Conclusion

In this review, we found that proper selenium intake, especially supplementary selenium, has a protective effect against multiple diseases. Selenium consumption in adults may reduce the risk of several digestive system cancers (including gastrointestinal, liver, and pancreatic cancers), all-cause mortality, depression, and Keshan disease in children, which in turn reduces the risk of Kashin-Beck disease. Furthermore, selenium supplementation improves sperm quality, PCOS, ATID, CHD, and infective outcomes. However, to date, no evidence has shown that selenium is associated with better outcomes among patients in ICUs. Based on our study findings and owing to the adverse effects and limited advantages of selenium intake, it is not recommended to receive extra supplementary selenium for general populations, and selenium supplementation should not be continued in patients whose selenium-deficient status has been corrected. Moreover, specific optimal dosage of selenium intake for general populations is still hard to determined due to the various between different areas. Thus, further large-scale high-quality prospective studies are required to confirm our findings.

## Author contributions

PW: Conceptualization, Data curation, Formal analysis, Investigation, Methodology, Project administration, Supervision, Writing – original draft. BC: Conceptualization, Data curation, Investigation, Formal analysis, Methodology, Writing – review & editing. YH: Writing – review & editing. JL: Writing – review & editing. DC: Writing – review & editing. ZC: Writing – review & editing. JZL: Conceptualization, Writing – review & editing. BR: Writing – review & editing. JY: Writing – review & editing. RW: Writing – review & editing. QW: Writing – review & editing. QD: Writing – review & editing. LL: Writing – review & editing.

## Funding

The author(s) declare financial support was received for the research, authorship, and/or publication of this article. This study was supported by Natural Science Foundation of China (Grant No. 82000721).

## Conflict of interest

The authors declare that the research was conducted in the absence of any commercial or financial relationships that could be construed as a potential conflict of interest.

## Publisher’s note

All claims expressed in this article are solely those of the authors and do not necessarily represent those of their affiliated organizations, or those of the publisher, the editors and the reviewers. Any product that may be evaluated in this article, or claim that may be made by its manufacturer, is not guaranteed or endorsed by the publisher.

## Figures and Tables

**Table:** 

RCT	randomized controlled trials
NRCT	non-randomized controlled trials
SMD	standard mean difference
WMD	weighted mean difference
OR	odds ratio
RR	relative risk
CI	confidence intervals
GRADE	the Grading of Recommendations, Assessment, Development and Evaluation
ATID	autoimmune thyroid disease
FT3	free triiodothyronine
FT4	free thyroxine
TSH	thyroid stimulating hormone
LT4	levothyroxine
TGAb	anti-thyroglobulin antibody
TPOAb	anti-thyroid peroxidase antibody
MMI	methimazole
HOMA-B	homeostasis model of assessment-estimated β-cell function
HOMA-IR	homeostasis model of assessment-estimated insulin resistance
QUICKI	quantitative insulin sensitivity check index
FPG	fasting plasma glucose
HbA1c	hemoglobin A1c
PCOS	polycystic ovary syndrome
TAC	total antioxidant capacity
SHBG	sex hormone binding globulin
TC	total cholesterol
VLDL	very low density lipoprotein-cholesterol
SBP	systolic blood pressure
DBP	diastolic blood pressure
TG	total triglyceride
LDL-C	lipoprotein-cholesterol
HDL-C	high-density lipoprotein-cholesterol
BMI	body mass index
ICU	intensive care units
hs-CRP	high sensitivity c-reactive protein
CRP	c-reactive protein
IQR	interquartile-range
CVD	cardiovascular disease
RDAs	recommended dietary allowances
GPXs	glutathione peroxidases
DIOs	iodothyronine deiodinases
SeMet	selenomethionine
ATP	adenosine triphosphate
PSTK	O-phosphoseryl-tRNA[Ser]Sec kinase
SEPHS2	selenophosphate synthetase 2
SEPSECS	Sec-tRNA synthase
SECIS	selenocysteine insertion sequence
ROS	reactive oxygen species
SELENOW	Selenoprotein W
SELENOT	Selenoprotein T
SELENOP	Selenoprotein P
NK cells	natural kill cells
IL	Interleukin
AIDS	acquired immune deficiency syndrome
COVID-19	corona virus disease-19
NF-κB	nuclear factor-kappa B
SNPs	single nucleotide polymorphisms
TR	thioredoxin reductase
CVB3	coxsackie virus B3
GCLC	γ-glutamate cysteine ligase
