# Supplementary material for: Selenium intake and multiple health-related outcomes: an umbrella review of meta-analyses
Source: Front Nutr. 2023 Sep 13;10:1263853. doi: 10.3389/fnut.2023.1263853 (PMC10534049; doi:10.3389/fnut.2023.1263853)
Supplement: Supplementary file 1 [file Table_1.docx]

| Outcome | Author-Year | Type | Population | No. of cases/total | Metrics | Estimates | 95%CI | No. of studies | Cohort | Case control | Cross-sectional | RCT | Effects model | I^2^ | Q test p value | Egger test p value |
| --- | --- | --- | --- | --- | --- | --- | --- | --- | --- | --- | --- | --- | --- | --- | --- | --- |
| Endocrine and metabolism outcomes |  |  |  |  |  |  |  |  |  |  |  |  |  |  |  |  |
| *Significant associations* |  |  |  |  |  |  |  |  |  |  |  |  |  |  |  |  |
| ATID |  |  |  |  |  |  |  |  |  |  |  |  |  |  |  |  |
| *Total ATID* |  |  |  |  |  |  |  |  |  |  |  |  |  |  |  |  |
| FT3 | Zuo, 2021 | Supplement | Adults | 338/659 | SMD^a^ | -0.40 | -0.70, -0.10 | 8 | 0 | 0 | 0 | 8 | Random | 92 | <0.00001 | NA |
| FT4 | Zuo, 2021 | Supplement | Adults | 427/845 | SMD^a^ | -0.76 | -1.58, -0.07 | 8 | 0 | 0 | 0 | 8 | Random | 89 | <0.00001 | NA |
| TPOAb(3 month) | Wichman, 2016 | Supplement | Adults | NA/1494 | WMD^a^ | -271 | -366, -175 | 7 | 0 | 0 | 0 | 7 | Random | 45.4 | <0.0001 | NA |
| TPOAb(6 month) | Wichman, 2016 | Supplement | Adults | NA/1494 | WMD^a^ | -469 | -617, -322 | 3 | 0 | 0 | 0 | 3 | Random | 52.4 | <0.00001 | NA |
| TPOAb(9 month) | Wichman, 2016 | Supplement | Adults | NA/1494 | WMD^a^ | -423 | -450, -396 | 1 | 0 | 0 | 0 | 1 | Random | NA | - | NA |
| TGAb(9 month) | Wichman, 2016 | Supplement | Adults | NA/1494 | WMD^a^ | -176 | -199, -153 | 1 | 0 | 0 | 0 | 1 | Random | NA | - | NA |
| *Hashimoto's thyroiditis* |  |  |  |  |  |  |  |  |  |  |  |  |  |  |  |  |
| well-being/mood | Toulis, 2010 | Supplement | Adults | 136/259 | RR^b^ | 2.79 | 1.21, 6.47 | 3 | 0 | 0 | 0 | 3 | Random | 75.6 | 0.017 | NA |
| TPOAb | Toulis, 2010 | Supplement | Adults | 136/259 | WMD^b^ | -271.09 | -421.98, -120.19 | 4 | 0 | 0 | 0 | 4 | Random | 62.6 | 0.046 | 0.235 |
| *Graves’ disease* |  |  |  |  |  |  |  |  |  |  |  |  |  |  |  |  |
| FT3(3 months) | Zheng, 2018 | Supplement | Adults | NA/736 | SMD^a^ | -0.34 | -0.66, -0.02 | 2 | 0 | 0 | 0 | 2 | Random | 0 | 0.719 | >0.05 |
| FT3(6 months) | Zheng, 2018 | Supplement | Adults | NA/736 | SMD^a^ | -0.67 | -0.97,- 0.36 | 4 | 0 | 0 | 0 | 4 | Random | 27.1 | 0.249 | >0.05 |
| FT4(3 months) | Zheng, 2018 | Supplement | Adults | NA/736 | SMD^a^ | -0.86 | -1.20, -0.53 | 2 | 0 | 0 | 0 | 2 | Random | 0 | 0.756 | >0.05 |
| FT4(6 months) | Zheng, 2018 | Supplement | Adults | NA/736 | SMD^a^ | -1.01 | -1.43, -0.60 | 4 | 0 | 0 | 0 | 4 | Fixed | 57.4 | 0.071 | >0.05 |
| TSH(6 months) | Zheng, 2018 | Supplement | Adults | NA/651 | SMD^a^ | 3.12 | 1.73, 4.5 | 3 | 0 | 0 | 0 | 3 | Fixed | 90.3 | 0 | >0.05 |
| Type 2 diabetes |  |  |  |  |  |  |  |  |  |  |  |  |  |  |  |  |
| incidence | Vinceti, 2018 | Supplement | Adults | 11,469/22,265 | RR^c^ | 1.11 | 1.01, 1.22 | 5 | 0 | 0 | 0 | 5 | Random | 0 | NA | NA |
| incidence | Vinceti, 2021 | Diet | Adults | NA/98,336 | RR^d^ | 1.55 | 1.27, 1.90 | 6 | 3 | 2 | 1 | 0 | Random | NA | NA | NA |
| HOMA-B | Gorabi, 2020 | Supplement | Adults | 130/260 | SMD^a^ | −0.63 | −0.89, -0.38 | 4 | 0 | 0 | 0 | 4 | Fixed | 0 | 0.75 | 0.17 |
| HOMA-IR | Ouyang. 2022 | Supplement | Adults | NA/526 | SMD^a^ | −0.50 | −0.86, -0.14 | 10 | 0 | 0 | 0 | 10 | Random | 75 | <0.00001 | 0.908 |
| QUIKI | Gorabi, 2020 | Supplement | Adults | 130/260 | SMD^a^ | 0.74 | 0.49, 0.1 | 4 | 0 | 0 | 0 | 4 | Fixed | 0 | 0.91 | 0.58 |
| Metabolic Syndrome | Ding, 2022 | Diet | Adults | NA/6,746 | RR^e^ | 0.77 | 0.63, 0.95 | 4 | 0 | 0 | 4 | 0 | Random | 0 | 0.985 | 1.000 |
| *Insignificant associations* |  |  |  |  |  |  |  |  |  |  |  |  |  |  |  |  |
| ATID |  |  |  |  |  |  |  |  |  |  |  |  |  |  |  |  |
| *Total ATID* |  |  |  |  |  |  |  |  |  |  |  |  |  |  |  |  |
| TSH | Zuo, 2021 | Supplement | Adults | 437/851 | SMD^a^ | 0.06 | −0.53, 0.66 | 8 | 0 | 0 | 0 | 8 | Random | 94 | <0.00001 | NA |
| TGAb(3 month) | Wichman, 2016 | Supplement | Adults | NA/1494 | WMD^a^ | 26 | -251, 304 | 6 | 0 | 0 | 0 | 6 | Random | 91.7 | 0.85 | NA |
| TGAb(6 month) | Wichman, 2016 | Supplement | Adults | NA/1494 | WMD^a^ | -111 | -252, 30 | 3 | 0 | 0 | 0 | 3 | Random | 0 | NA | NA |
| *Graves’ disease* |  |  |  |  |  |  |  |  |  |  |  |  |  |  |  |  |
| FT3(9 months) | Zheng, 2018 | Supplement | Adults | NA/736 | SMD^a^ | 0.01 | -0.44, 0.46 | 3 | 0 | 0 | 0 | 5 | Fixed | 65.8 | 0.054 | >0.05 |
| FT4(9 months) | Zheng, 2018 | Supplement | Adults | NA/736 | SMD^a^ | 0.03 | -0.29, 0.35 | 3 | 0 | 0 | 0 | 5 | Random | 38.9 | 0.195 | >0.05 |
| TSH(3 months) | Zheng, 2018 | Supplement | Adults | NA/651 | SMD^a^ | 0.21 | -0.15, 0.57 | 1 | 0 | 0 | 0 | 1 | - | - | - | >0.05 |
| TSH(9 months) | Zheng, 2018 | Supplement | Adults | NA/651 | SMD^a^ | -2.27 | -4.74, 0.21 | 3 | 0 | 0 | 0 | 5 | Fixed | 97.8 | 0 | >0.05 |
| TRAb(6 months) | Zheng, 2018 | Supplement | Adults | NA/736 | SMD^a^ | -2.31 | -4.63, 0.00 | 3 | 0 | 0 | 0 | 3 | Fixed | 97.1 | 0 | >0.05 |
| TRAb(9 months) | Zheng, 2018 | Supplement | Adults | NA/736 | SMD^a^ | -0.49 | -1.82, 0.83 | 3 | 0 | 0 | 0 | 3 | Fixed | 95.8 | 0 | >0.05 |
| Type 2 diabetes |  |  |  |  |  |  |  |  |  |  |  |  |  |  |  |  |
| Insulin level | Gorabi, 2020 | Supplement | Adults | 256/499 | SMD^a^ | −0.23 | −0.6, 0.14 | 9 | 0 | 0 | 0 | 9 | Random | 76.6 | <0.001 | 0.50 |
| FPG | Gorabi, 2020 | Supplement | Adults | 275/535 | SMD^a^ | −0.03 | −0.4, 0.34 | 9 | 0 | 0 | 0 | 9 | Random | 78.4 | <0.001 | 0.37 |
| HbA1c | Gorabi, 2020 | Supplement | Adults | 96/178 | SMD^a^ | 0.1 | −0.63, 0.84 | 3 | 0 | 0 | 0 | 3 | Random | 83.1 | 0.003 | 0.7 |
|  |  |  |  |  |  |  |  |  |  |  |  |  |  |  |  |  |
| Mental and cognitive outcomes |  |  |  |  |  |  |  |  |  |  |  |  |  |  |  |  |
| *Significant associations* |  |  |  |  |  |  |  |  |  |  |  |  |  |  |  |  |
| Depression |  |  |  |  |  |  |  |  |  |  |  |  |  |  |  |  |
| *total depression* | Ding, 2022 | Diet | Adults | NA/37,497 | RR^e^ | 0.63 | 0.54, 0.74 | 6 | 2 | 0 | 4 | 0 | Random | 37.8 | 0.154 | 0.260 |
| *Postpartum depression* | Sajjadi, 2022 | Supplement | Adults | NA/638 | OR^e^ | 0.97 | 0.95, 0.99 | 2 | 1 | 1 | 0 | 0 | Random | 0 | 0.507 | 0.65 |
| *Depression scores* | Sajjadi, 2022 | Supplement | Adults | NA/310 | WMD^e^ | −0.37 | −0.56, -0.18 | 3 | 0 | 0 | 1 | 2 | Random | 0 | 0.959 | 0.11 |
| *Insignificant associations* |  |  |  |  |  |  |  |  |  |  |  |  |  |  |  |  |
| Depression |  |  |  |  |  |  |  |  |  |  |  |  |  |  |  |  |
| *Other types of depression* | Sajjadi, 2022 | Supplement | Adults | NA/44,030 | OR^e^ | 1.06 | 0.75, 1.50 | 5 | 1 | 1 | 3 | 0 | Random | 85.6 | <0.001 | 0.65 |
|  |  |  |  |  |  |  |  |  |  |  |  |  |  |  |  |  |
| Reproductive outcomes |  |  |  |  |  |  |  |  |  |  |  |  |  |  |  |  |
| *Significant associations* |  |  |  |  |  |  |  |  |  |  |  |  |  |  |  |  |
| Male Infertility |  |  |  |  |  |  |  |  |  |  |  |  |  |  |  |  |
| Sperm concentration | Sharma, 2022 | Supplement | Adults | 176/329 | MD^a^ | 6.58 | 3.22, 9.93 | 3 | 0 | 0 | 0 | 3 | Random | 70 | 0.04 | NA |
| Semen Volume | Sharma, 2022 | Supplement | Adults | 105/211 | MD^a^ | 0.40 | 0.01, 0.79 | 1 | 0 | 0 | 0 | 1 | Random | - | - | - |
| Sperm motility | Salas-Huetos, 2018 | Supplement | Adults | 143/287 | MD^b^ | 3.30 | 2.95, 3.65 | 3 | 0 | 0 | 0 | 3 | Random | 20 | 0.29 | NA |
| Sperm morphology | Salas-Huetos, 2018 | Supplement | Adults | 127/253 | MD^b^ | 1.87 | 1.50, 2.24 | 2 | 0 | 0 | 0 | 2 | Random | 0 | 0.34 | NA |
| Polycystic ovary syndrome |  |  |  |  |  |  |  |  |  |  |  |  |  |  |  |  |
| SHBG | Zhao, 2023 | Supplement | Adults | 90/179 | WMD^b^ | 9.747 | 0.32, 19.18 | 3 | 0 | 0 | 0 | 3 | Random | 0 | 0.952 | 0.075 |
| total testosterone | Wu, 2022 | Supplement | Adults | NA | SMD^b^ | -0.42 | -0.78, -0.06 | 5 | 0 | 0 | 0 | 5 | Random | NA | NA | NA |
| cholesterol | Wu, 2022 | Supplement | Adults | NA | SMD^b^ | -0.71 | -1.41, -0.02 | 5 | 0 | 0 | 0 | 5 | Random | NA | NA | NA |
| *Insignificant associations* |  |  |  |  |  |  |  |  |  |  |  |  |  |  |  |  |
| Male Infertility |  |  |  |  |  |  |  |  |  |  |  |  |  |  |  |  |
| Pregnancy Rate | Sharma, 2022 | Supplement | Adults | 41/61 | OR^a^ | 5.27 | 0.28, 100.83 | 1 | 0 | 0 | 0 | 1 | Random | - | - | - |

**Supplementary Table 1. Associations between selenium intake and endocrine, metabolism, mental, cognitive and reproductive outcomes.**

CI, confidence interval; MD, mean difference; NA, not available; OR, odds ratio; RCT, randomized controlled trial; RR, relative risk; SMD, standardize mean difference; WMD, weighted mean difference. ATID, autoimmune thyroid disease; FT3, serum free triiodothyronine; FT4, Serum free thyroxine; TPOAb, anti-thyroid peroxidase antibody; TGAb, anti-thyroglobulin antibody; TRAb, thyrotrophic hormone receptor antibody; TSH, thyroid stimulating hormone; FPG, fasting plasma glucose; HbA1c, Hemoglobin A1c; HOMA-B, assessment-estimated β-cell function; QUICKI, quantitative insulin sensitivity check index; HOMA-IR, assessment-estimated insulin resistance; SHBG, sex hormone binding globulin.

^a^ combination of selenium versus placebo or not

^b^ Selenium 100 µg or 200 µg supplementation versus not

^c^ 200 μg/day selenium intake

^d^ 120μg/day versus 55μg/day

^e^ highest versus lowest.
