# Supplementary material for: Selenium intake and multiple health-related outcomes: an umbrella review of meta-analyses
Source: Front Nutr. 2023 Sep 13;10:1263853. doi: 10.3389/fnut.2023.1263853 (PMC10534049; doi:10.3389/fnut.2023.1263853)
Supplement: Supplementary file 2 [file Table_2.docx]

| Outcome | Author-Year | Type | Population | No. of cases/total | Metrics | Estimates | 95%CI | No. of studies | Cohort | Case control | Cross-sectional | RCT | Effects model | I^2^ | Q test p value | Egger test p value |
| --- | --- | --- | --- | --- | --- | --- | --- | --- | --- | --- | --- | --- | --- | --- | --- | --- |
| Circulatory outcomes |  |  |  |  |  |  |  |  |  |  |  |  |  |  |  |  |
| *Significant associations* |  |  |  |  |  |  |  |  |  |  |  |  |  |  |  |  |
| Coronary heart disease |  |  |  |  |  |  |  |  |  |  |  |  |  |  |  |  |
| TC | Kelishadi, 2022 | Supplement | Adults | 1951/2984 | WMD^a^ | -2.11 | -4.09, -0.13 | 19 | 0 | 0 | 0 | 19 | Fixed | 0 | 0.645 | 0.511 |
| VLDL-C | Kelishadi, 2022 | Supplement | Adults | 1951/2984 | WMD^a^ | -1.35 | −2.33, −0.37 | 8 | 0 | 0 | 0 | 8 | Fixed | 0 | 0.488 | 0.932 |
| SBP | Kelishadi, 2022 | Supplement | Adults | 1951/2984 | WMD^a^ | 2.02 | 0.50, 3.55 | 5 | 0 | 0 | 0 | 5 | Random | 23.2 | 0.267 | 0.317 |
| Keshan Disease | Zhou, 2018 | Supplement | Adults | 683,075/1,983,238 | RR^f^ | 0.14 | 0.12, 0.16 | 17 | NA | NA | 0 | 0 | Fixed | 41.8 | < 0.05 | 0.285 |
| *Insignificant associations* |  |  |  |  |  |  |  |  |  |  |  |  |  |  |  |  |
| Coronary heart disease |  |  |  |  |  |  |  |  |  |  |  |  |  |  |  |  |
| Mortality | Ju, 2017 | Supplement | Adults | 756/4,1763 | OR^a^ | 0.88 | 0.76, 1.02 | 8 | 0 | 0 | 0 | 8 | Random | 46.9 | 0.068 | NA |
| All CVD events | Rees, 2013 | Supplement | Adults | NA | RR^a^ | 1.03 | 0.95, 1.11 | 2 | 0 | 0 | 0 | 2 | Random | NA | NA | NA |
| TG | Kelishadi, 2022 | Supplement | Adults | 1951/2984 | WMD^a^ | -0.85 | -4.74, 3.06 | 19 | 0 | 0 | 0 | 19 | Random | 16.8 | 0.230 | 0.788 |
| LDL-C | Kelishadi, 2022 | Supplement | Adults | 1951/2984 | WMD^a^ | 0.86 | −1.21, 2.95 | 17 | 0 | 0 | 0 | 17 | Random | 34.5 | 0.062 | 0.067 |
| HDL-C | Kelishadi, 2022 | Supplement | Adults | 1951/2984 | WMD^a^ | 0.30 | −0.66, 1.27 | 21 | 0 | 0 | 0 | 17 | Random | 61.5 | < 0.001 | 0.433 |
| DBP | Kelishadi, 2022 | Supplement | Adults | 1951/2984 | WMD^a^ | 0.39 | −0.89, 1.68 | 5 | 0 | 0 | 0 | 5 | Fixed | 0.0 | 0.627 | 0.813 |
| BMI | Kelishadi, 2022 | Supplement | Adults | 1951/2984 | WMD^a^ | 0.01 | −0.04, 0.07 | 8 | 0 | 0 | 0 | 8 | Fixed | 0.0 | 0.896 | NA |
|  |  |  |  |  |  |  |  |  |  |  |  |  |  |  |  |  |
| Skeletel outcomes |  |  |  |  |  |  |  |  |  |  |  |  |  |  |  |  |
| *Significant associations* |  |  |  |  |  |  |  |  |  |  |  |  |  |  |  |  |
| Kashin-Beck disease |  |  |  |  |  |  |  |  |  |  |  |  |  |  |  |  |
| Radiographic improvement | Zou, 2019 | Supplement | Children | NA/9815 | RR^a^ | 1.88 | 1.51, 2.33 | 22 | 0 | 0 | 0 | 22 | Random | NA | NA | NA |
| Risk | Zou, 2008 | Supplement | Children | 189/409 | OR^a^ | 0.13 | 0.04, 0.47 | 4 | 0 | 0 | 0 | 4 | Random | 0 | 0.89 | NA |
|  |  |  |  |  |  |  |  |  |  |  |  |  |  |  |  |  |
| Critical-ill outcomes |  |  |  |  |  |  |  |  |  |  |  |  |  |  |  |  |
| *Insignificant associations* |  |  |  |  |  |  |  |  |  |  |  |  |  |  |  |  |
| Total mortality | Manzanares, 2016 | Supplement | Adult | 1,182/2,371 | RR^c^ | 0.98 | 0.90, 1.08 | 21 | 0 | 0 | 0 | 21 | Random | 12 | 0.33 | 0.08 |
| New infectious complications | Manzanares, 2016 | Supplement | Adult | 1,182/2,371 | RR^c^ | 0.95 | 0.88, 1.02 | 4 | 0 | 0 | 0 | 4 | Random | 0 | 0.34 | 0.19 |
| Hospital stay | Mousavi, 2021 | Supplement | Adult | 1,317/2,909 | WMD^c^ | 0.84 | -6.92, 13.05 | 8 | 0 | 0 | 0 | 8 | Random | 97.52 | < 0.001 | NA |
| ICU stay | Mousavi, 2021 | Supplement | Adult | 1,300//2,937 | WMD^c^ | 0.19 | -0.33, 0.71 | 11 | 0 | 0 | 0 | 11 | Random | 97.97 | < 0.001 | NA |
| New renal dysfunction | Manzanares, 2016 | Supplement | Adult | NA/2,371 | RR^c^ | 0.79 | 0.57, 1.08 | 10 | 0 | 0 | 0 | 10 | Random | 0 | NA | NA |
| Survival |  |  |  |  |  |  |  |  |  |  |  |  |  |  |  |  |
| 28 day | Mousavi, 2021 | Supplement | Adult | 1,601/3,430 | OR^c^ | 1.08 | 0.87, 1.34 | 19 | 0 | 0 | 0 | 19 | Random | 24 | 0.16 | p>0.05 |
| 3 months | Mousavi, 2021 | Supplement | Adult | 449/899 | OR^c^ | 1.18 | 0.68, 2.04 | 3 | 0 | 0 | 0 | 3 | Random | 63.4 | 0.06 | p>0.05 |
| 6 months | Mousavi, 2021 | Supplement | Adult | 251/478 | OR^c^ | 1.29 | 0.89, 1.86 | 2 | 0 | 0 | 0 | 2 | Random | 75 | 0.3 | p>0.05 |
| Ventilator days | Manzanares, 2016 | Supplement | Adult | NA/2,371 | WMD^c^ | -1.75 | -4.30, 0.81 | 8 | 0 | 0 | 0 | 8 | Random | 74 | 0.0004 | NA |
|  |  |  |  |  |  |  |  |  |  |  |  |  |  |  |  |  |
| Infective outcomes |  |  |  |  |  |  |  |  |  |  |  |  |  |  |  |  |
| *Significant associations* |  |  |  |  |  |  |  |  |  |  |  |  |  |  |  |  |
| Spesis |  |  |  |  |  |  |  |  |  |  |  |  |  |  |  |  |
| *Duration of vasopressor therapy* | Li, 2019 | Supplement | Adult | 621/1,246 | SMD^c^ | -0.75 | -1.37, -0.13 | 4 | 0 | 0 | 0 | 4 | Random | 87 | < .0001 | NA |
| *Respiratory tract infections* | Nazari, 2022 | Supplement | Adult | NA | OR^c^ | 0.62 | 0.545, 0.696 | 1 | 0 | 1 | 0 | 0 | Random | NA | NA | NA |
| Inflammatory markers |  |  |  |  |  |  |  |  |  |  |  |  |  |  |  |  |
| hs-CRP | Djalalinia, 2021 | Supplement | Adult | 150/298 | SMD^c^ | -0.44 | -0.67,-0.21 | 5 | 0 | 0 | 0 | 5 | Fixed | 12.6 | 0.33 | 0.18 |
| CRP | Djalalinia, 2021 | Supplement | Adult | 43/84 | SMD^c^ | 0.6 | 0.16, 1.04 | 2 | 0 | 0 | 0 | 2 | Random | 73.2 | < 0.001 | 0.18 |
| *Insignificant associations* |  |  |  |  |  |  |  |  |  |  |  |  |  |  |  |  |
| Spesis |  |  |  |  |  |  |  |  |  |  |  |  |  |  |  |  |
| *Mortality* |  |  |  |  |  |  |  |  |  |  |  |  |  |  |  |  |
| 28 days | Li, 2019 | Supplement | Adult | 961/1,922 | RR^c^ | 0.94 | 0.82, 1.06 | 13 | 0 | 0 | 0 | 13 | Fixed | 0 | 0.47 | NA |
| 3 months | Li, 2019 | Supplement | Adult | 572/1,143 | RR^c^ | 0.73 | 0.36, 1.47 | 2 | 0 | 0 | 0 | 2 | Random | 83 | 0.01 | NA |
| 6 months | Li, 2019 | Supplement | Adult | 31/50 | RR^c^ | 1.16 | 0.78, 1.71 | 1 | 0 | 0 | 0 | 1 | Random | NA | NA | NA |
| *New renal dysfunction* | Li, 2019 | Supplement | Adult | 114/227 | RR^c^ | 0.65 | 0.41, 1.03 | 5 | 0 | 0 | 0 | 5 | Fixed | 0 | 0.59 | NA |
| *Secondary infection* | Li, 2019 | Supplement | Adult | 734/1,472 | RR^c^ | 0.96 | 0.87, 1.06 | 5 | 0 | 0 | 0 | 5 | Fixed | 38 | 0.17 | NA |
| Inflammatory markers |  |  |  |  |  |  |  |  |  |  |  |  |  |  |  |  |
| NO | Djalalinia, 2021 | Supplement | Adult | 114/228 | SMD^c^ | 0.003 | -0.26, 0.26 | 4 | 0 | 0 | 0 | 4 | Fixed | 28.4 | 0.24 | 0.18 |
|  |  |  |  |  |  |  |  |  |  |  |  |  |  |  |  |  |
| Other outcomes |  |  |  |  |  |  |  |  |  |  |  |  |  |  |  |  |
| *Significant associations* |  |  |  |  |  |  |  |  |  |  |  |  |  |  |  |  |
| Preeclampsia | Xu, 2015 | Supplement | Adult | NA/439 | RR^b^ | 0.28 | 0.09, 0.84 | 3 | 0 | 0 | 0 | 3 | Random | 0 | 0.63 | 0.048 |
| *Insignificant associations* |  |  |  |  |  |  |  |  |  |  |  |  |  |  |  |  |
| Immune function |  |  |  |  |  |  |  |  |  |  |  |  |  |  |  |  |
| IgA | Filippini, 2022 | Supplement | Adult | NA/370 | SMD^cd^ | 0.13 | -0.16, 0.42 | 3 | 0 | 0 | 0 | 3 | Random | 0 | NA | NA |
| IgG | Filippini, 2022 | Supplement | Adult | NA/370 | SMD^c^ | 0.14 | −0.97, 1.25 | 2 | 0 | 0 | 0 | 2 | Random | 65.64 | NA | NA |
| IgM | Filippini, 2022 | Supplement | Adult | NA/370 | SMD^c^ | 0.09 | −0.50, 0.67 | 2 | 0 | 0 | 0 | 2 | Random | 0 | NA | NA |
| NK cells overall levels | Filippini, 2022 | Supplement | Adult | NA/370 | SMD^ce^ | -0.14 | -0.49, 0.21 | 2 | 0 | 0 | 0 | 2 | Random | 30.48 | NA | NA |
| lymphocyte overall levels | Filippini, 2022 | Supplement | Adult | NA/370 | SMD^c^ | -0.25 | -0.77, 0.27 | 4 | 0 | 0 | 0 | 4 | Random | 0 | NA | NA |
|  |  |  |  |  |  |  |  |  |  |  |  |  |  |  |  |  |

**Supplementary Table 2. Associations between selenium intake and circulatory, skeletel, mental, critical-ill, infective and other outcomes.**

CI, confidence interval; NA, not available; OR, odds ratio; RCT, randomized controlled trial; RR, relative risk; SMD, standardize mean difference; WMD, weighted mean difference. TC, total cholesterol; TG, triglyceride; CVD, cardiovascular disease; LDL-C, low-density lipoprotein-cholesterol; HDL-C, high-density lipoprotein-cholesterol; VLDL-C, very low density lipoprotein-cholesterol; DBP, diastolic blood pressure; SBP, systolic blood pressure; BMI, Body Mass Index; ICU, intensive care units; CRP, c-reactive protein; hs-CRP, high sensitivity c-reactive protein; NO, nitric oxide. IgA, immunoglobulin A; IgG, immunoglobulin G; IgM, immunoglobulin M, NK cells, natural kill cells.

^a^ combination of selenium versus placebo or not

^b^ Selenium 60 µg or 100 µg supplementation versus not

^c^ highest versus lowest.

^d^ dose-response meta-analysis presents selenium increase above 40µg/L is associated with higher IgA levels until 110 µg/L.

^e^ dose-response meta-analysis presents plasma selenium is associated with an increase in NK cells until 140 µg/L.

^f^ ever use versus not.
