# Supplementary material for: Selenium intake and multiple health-related outcomes: an umbrella review of meta-analyses
Source: Front Nutr. 2023 Sep 13;10:1263853. doi: 10.3389/fnut.2023.1263853 (PMC10534049; doi:10.3389/fnut.2023.1263853)
Supplement: Supplementary file 3 [file Table_3.docx]

| Supplementary Table 3. Assessments of AMSTAR^a^ scores for mortality and cancer outcomes of included meta-analyses. | | | | | | | | | | | | | | |
| --- | --- | --- | --- | --- | --- | --- | --- | --- | --- | --- | --- | --- | --- | --- |
| Outcome | Assessed with | Author and year | A priori design provided | Duplicate study selection & data extraction | At least two electronic databases searched | Status of  publication used as an inclusion criterion | List of  included and excluded studies provided | Characteristics of  included studies provided | Scientific quality of  included studies assessed | Scientific quality of  the included studies  Used appropriately to form conclusions | Appropriate methods to  combine studies | Publication bias assessed | Conflict of interest included | Total AMSTAR Score |
| ***Mortality outcomes*** |  |  |  |  |  |  |  |  |  |  |  |  |  |  |
| All-cause mortality | Highest versus lowest | Jayedi, 2018 | 1 | 1 | 1 | 0 | 0 | 1 | 1 | 0 | 1 | 0 | 0 | 6 |
| All-cause mortality | Highest versus lowest | Bjelakovic, 2007 | 1 | 1 | 1 | 0 | 0 | 1 | 0 | 0 | 1 | 0 | 1 | 6 |
| ***Cancer outcomes*** |  |  |  |  |  |  |  |  |  |  |  |  |  |  |
| All cancer | Highest versus lowest | Lee EH, 2009 | 1 | 1 | 1 | 0 | 0 | 1 | 0 | 0 | 1 | 0 | 1 | 6 |
| All cancer | >55 μg/day versus never | Kuria A, 2020 | 1 | 1 | 1 | 0 | 0 | 1 | 1 | 1 | 1 | 0 | 1 | 8 |
| Gastrointestinal cancer | All dose versus never | Bjelakovic G, 2004 | 1 | 1 | 1 | 0 | 0 | 1 | 0 | 0 | 0 | 1 | 1 | 6 |
| Liver cancer | Highest versus lowest | Vinceti M, 2018 | 1 | 1 | 1 | 0 | 0 | 1 | 1 | 1 | 0 | 1 | 1 | 8 |
| Liver cancer | All dose versus never | Kuria A, 2020 | 1 | 1 | 1 | 0 | 0 | 1 | 1 | 1 | 1 | 0 | 1 | 8 |
| Pancreatic cancer | Highest versus lowest | Wang L, 2016 | 1 | 1 | 1 | 0 | 0 | 1 | 1 | 0 | 1 | 1 | 1 | 8 |
| Pancreatic cancer | Highest versus lowest | Chen J, 2016 | 1 | 1 | 1 | 0 | 0 | 1 | 1 | 0 | 0 | 1 | 1 | 7 |
| Skin cancer | >55 μg/day versus never | Kuria A, 2020 | 1 | 1 | 1 | 0 | 0 | 1 | 1 | 1 | 1 | 0 | 1 | 8 |
| Breast cancer | Highest versus lowest | Vinceti M, 2018 | 1 | 1 | 1 | 0 | 0 | 1 | 1 | 1 | 0 | 1 | 1 | 8 |
| Breast cancer | Highest versus lowest | Kuria A, 2020 | 1 | 1 | 1 | 0 | 0 | 1 | 1 | 1 | 1 | 0 | 1 | 8 |
| Head and neck cancer | Highest versus lowest | Vinceti M, 2018 | 1 | 1 | 1 | 0 | 0 | 1 | 1 | 1 | 0 | 1 | 1 | 8 |
| Colorectal cancer | Highest versus lowest | Vinceti M, 2018 | 1 | 1 | 1 | 0 | 0 | 1 | 1 | 1 | 0 | 1 | 1 | 8 |
| Colorectal cancer | all dose versus never | Kuria A, 2020 | 1 | 1 | 1 | 0 | 0 | 1 | 1 | 1 | 1 | 0 | 1 | 8 |
| Esophageal cancer | Highest versus lowest | Vinceti M, 2018 | 1 | 1 | 1 | 0 | 0 | 1 | 1 | 1 | 0 | 1 | 1 | 8 |
| Esophageal cancer | 10 μg/day selenium intake increase | Hong B, 2016 | 1 | 1 | 1 | 0 | 0 | 1 | 1 | 0 | 1 | 1 | 0 | 7 |
| Melanoma | Highest versus lowest | Vinceti M, 2018 | 1 | 1 | 1 | 0 | 0 | 1 | 1 | 1 | 0 | 1 | 1 | 8 |
| Non-melanoma skin cancer | Highest versus lowest | Vinceti M, 2018 | 1 | 1 | 1 | 0 | 0 | 1 | 1 | 1 | 0 | 1 | 1 | 8 |
| Lung cancer | Highest versus lowest | Vinceti M, 2018 | 1 | 1 | 1 | 0 | 0 | 1 | 1 | 1 | 0 | 1 | 1 | 8 |
| Lung cancer | all dose versus never | Kuria A, 2020 | 1 | 1 | 1 | 0 | 0 | 1 | 1 | 1 | 1 | 0 | 1 | 8 |
| Bladder cancer | Highest versus lowest | Vinceti M, 2018 | 1 | 1 | 1 | 0 | 0 | 1 | 1 | 1 | 0 | 1 | 1 | 8 |
| Bladder cancer | Highest versus lowest | Kuria A, 2020 | 1 | 1 | 1 | 0 | 0 | 1 | 1 | 1 | 1 | 0 | 1 | 8 |
| Prostate cancer | Highest versus lowest | Sayehmiri K, 2018 | 1 | 1 | 1 | 0 | 0 | 1 | 1 | 0 | 1 | 1 | 1 | 8 |
| Prostate cancer | Highest versus lowest | Sayehmiri K, 2018 | 1 | 1 | 1 | 0 | 0 | 1 | 1 | 0 | 1 | 1 | 1 | 8 |
| ^a^: AMSTAR, a measurement tool to assess systematic reviews. | | | | | | | | | | | | | | |
