# Supplementary material for: Selenium intake and multiple health-related outcomes: an umbrella review of meta-analyses
Source: Front Nutr. 2023 Sep 13;10:1263853. doi: 10.3389/fnut.2023.1263853 (PMC10534049; doi:10.3389/fnut.2023.1263853)
Supplement: Supplementary file 5 [file Table_5.docx]

| Supplementary Table 5. GRADE classification of quality of evidence for mortality and cancer incidence. | | | | | | | | | | | | | | | | |
| --- | --- | --- | --- | --- | --- | --- | --- | --- | --- | --- | --- | --- | --- | --- | --- | --- |
| Outcome | Assessed with | Author | No. of  studies | Cohort | Case-control | RCT | NRCT | Risk of bias | Inconsistency | Indirectness | Imprecision | Publication bias | Plausible confounding | Magnitude of  effect | Dose-response gradient | Quality |
| ***Mortality outcomes*** |  |  |  |  |  |  |  |  |  |  |  |  |  |  |  |  |
| All-cause mortality | Highest versus lowest | Jayedi, 2018 | 3 | 3 | 0 | 0 | 0 | serious risk | no serious  inconsistency | no serious  indirectness | no serious  imprecision | undetected | would not  reduce effect | no | yes | moderate |
| All-cause mortality | Highest versus lowest | Bjelakovic, 2007 | 3 | 0 | 0 | 3 | 0 | serious risk | serious  inconsistency | no serious  indirectness | serious  imprecision | NA | would reduce effect | no | no | very low |
| ***Cancer outcomes*** |  |  |  |  |  |  |  |  |  |  |  |  |  |  |  |  |
| All cancer | Highest versus lowest | Lee EH, 2009 | 8 | 0 | 0 | 8 | 0 | serious risk | serious  inconsistency | no serious  indirectness | no serious  imprecision | undetected | would not  reduce effect | no | no | very low |
| All cancer | >55 μg/day versus never | Kuria A, 2020 | 106 | NA | NA | 0 | 0 | serious risk | no serious  inconsistency | no serious  indirectness | no serious  imprecision | NA | would reduce effect | no | yes | low |
| Gastrointestinal cancer | All dose versus never | Bjelakovic G, 2004 | 4 | 0 | 0 | 4 | 0 | serious risk | serious  inconsistency | no serious  indirectness | no serious  imprecision | NA | would not  reduce effect | no | no | very low |
| Liver cancer | Highest versus lowest | Vinceti M, 2018 | 4 | 0 | 0 | 4 | 0 | serious risk | no serious  inconsistency | no serious  indirectness | no serious  imprecision | undetected | would not  reduce effect | no | no | low |
| Liver cancer | All dose versus never | Kuria A, 2020 | 14 | NA | NA | 0 | 0 | serious risk | no serious  inconsistency | no serious  indirectness | no serious  imprecision | NA | would not  reduce effect | no | yes | low |
| Pancreatic cancer | Highest versus lowest | Wang L, 2016 | 6 | 3 | 3 | 0 | 0 | serious risk | no serious  inconsistency | no serious  indirectness | no serious  imprecision | undetected | would not reduce effect | no | no | low |
| Pancreatic cancer | Highest versus lowest | Chen J, 2016 | 6 | 3 | 3 | 0 | 0 | serious risk | serious  inconsistency | no serious  indirectness | no serious  imprecision | undetected | would reduce effect | no | no | low |
| Skin cancer | >55 μg/day versus never | Kuria A, 2020 | 21 | NA | NA | 0 | 0 | serious risk | no serious  inconsistency | no serious  indirectness | no serious  imprecision | NA | would not  reduce effect | no | yes | low |
| Breast cancer | Highest versus lowest | Vinceti M, 2018 | 3 | 0 | 0 | 3 | 0 | serious risk | no serious  inconsistency | no serious  indirectness | no serious  imprecision | undetected | would not  reduce effect | no | no | low |
| Breast cancer | Highest versus lowest | Kuria A, 2020 | 2 | NA | NA | 0 | 0 | serious risk | no serious  inconsistency | no serious  indirectness | no serious  imprecision | NA | would not  reduce effect | no | yes | low |
| Head and neck cancer | Highest versus lowest | Vinceti M, 2018 | 2 | 0 | 0 | 2 | 0 | serious risk | no serious  inconsistency | no serious  indirectness | no serious  imprecision | undetected | would not  reduce effect | no | no | low |
| Colorectal cancer | Highest versus lowest | Vinceti M, 2018 | 3 | 0 | 0 | 3 | 0 | serious risk | no serious  inconsistency | no serious  indirectness | no serious  imprecision | undetected | would not  reduce effect | no | no | low |
| Colorectal cancer | all dose versus never | Kuria A, 2020 | 14 | NA | NA | 0 | 0 | serious risk | no serious  inconsistency | no serious  indirectness | no serious  imprecision | NA | would not  reduce effect | no | yes | low |
| Esophageal cancer | Highest versus lowest | Vinceti M, 2018 | 2 | 0 | 0 | 2 | 0 | serious risk | no serious  inconsistency | no serious  indirectness | no serious  imprecision | undetected | would not  reduce effect | no | no | low |
| Esophageal cancer | 10 μg/day selenium intake increase | Hong B, 2016 | 4 | 1 | 3 | 0 | 0 | serious risk | serious  inconsistency | no serious  indirectness | no serious  imprecision | undetected | would not  reduce effect | no | yes | moderate |
| Melanoma | Highest versus lowest | Vinceti M, 2018 | 3 | 0 | 0 | 3 | 0 | serious risk | no serious  inconsistency | no serious  indirectness | no serious  imprecision | undetected | would not  reduce effect | no | no | low |
| Non-melanoma skin cancer | Highest versus lowest | Vinceti M, 2018 | 4 | 0 | 0 | 4 | 0 | serious risk | no serious  inconsistency | no serious  indirectness | no serious  imprecision | undetected | would not  reduce effect | no | no | low |
| Lung cancer | Highest versus lowest | Vinceti M, 2018 | 3 | 0 | 0 | 3 | 0 | serious risk | no serious  inconsistency | no serious  indirectness | no serious  imprecision | undetected | would not  reduce effect | no | no | low |
| Lung cancer | all dose versus never | Kuria A, 2020 | 4 | NA | NA | 0 | 0 | serious risk | no serious  inconsistency | no serious  indirectness | no serious  imprecision | NA | would reduce effect | no | yes | low |
| Bladder cancer | Highest versus lowest | Vinceti M, 2018 | 3 | 0 | 0 | 3 | 0 | serious risk | no serious  inconsistency | no serious  indirectness | no serious  imprecision | undetected | would not  reduce effect | no | no | low |
| Bladder cancer | Highest versus lowest | Kuria A, 2020 | 2 | NA | NA | 2 | NA | serious risk | serious  inconsistency | no serious  indirectness | no serious  imprecision | NA | would not  reduce effect | no | yes | low |
| Prostate cancer | Highest versus lowest | Sayehmiri K, 2018 | 9 | 0 | 0 | 9 | 0 | serious risk | no serious  inconsistency | no serious  indirectness | serious  imprecision | undetected | would reduce effect | no | no | very low |
| Prostate cancer | Highest versus lowest | Sayehmiri K, 2018 | 7 | 2 | 5 | 0 | 0 | serious risk | no serious  inconsistency | no serious  indirectness | serious  imprecision | strongly suspected | would reduce  reduce effect | no | no | very low |
| GRADE, Grading of Recommendations Assessment, Development, and Evaluation; RCT, randomized controlled trial; NRCT, non-randomized controlled trial; NA, not available. | | | | | | | | | | | | | | | | |
