# Supplementary material for: Selenium intake and multiple health-related outcomes: an umbrella review of meta-analyses
Source: Front Nutr. 2023 Sep 13;10:1263853. doi: 10.3389/fnut.2023.1263853 (PMC10534049; doi:10.3389/fnut.2023.1263853)
Supplement: Supplementary file 6 [file Table_6.docx]

| Supplementary Table 6. GRADE classification of quality of evidence for selenium intake and other health outcomes. | | | | | | | | | | | | | | | | |
| --- | --- | --- | --- | --- | --- | --- | --- | --- | --- | --- | --- | --- | --- | --- | --- | --- |
| Outcome | Assessed with | Author | No. of  studies | Cohort | Case-control | RCT | NRCT | Risk of bias | Inconsistency | Indirectness | Imprecision | Publication bias | Plausible confounding | Magnitude of  effect | Dose-response gradient | Quality |
| ***Endocrine and metabolism outcomes*** |  |  |  |  |  |  |  |  |  |  |  |  |  |  |  |  |
| ***Total ATID*** |  |  |  |  |  |  |  |  |  |  |  |  |  |  |  |  |
| FT3 | Combination of selenium and drugs versus placebo or not | Zuo, 2021 | 8 | 0 | 0 | 8 | 0 | serious risk | serious  inconsistency | no serious  indirectness | no serious imprecision | strongly suspected | would reduce effect | no | no | very low |
| FT4 | Combination of selenium and drugs versus placebo or not | Zuo, 2021 | 8 | 0 | 0 | 8 | 0 | serious risk | serious  inconsistency | no serious  indirectness | no serious imprecision | strongly suspected | would reduce effect | no | no | very low |
| TPOAb(3 month) | Combination of selenium and drugs versus placebo or not | Wichman, 2016 | 7 | 0 | 0 | 7 | 0 | serious risk | no serious  inconsistency | no serious  indirectness | no serious imprecision | NA | would not  reduce effect | no | no | low |
| TPOAb(6 month) | Combination of selenium and drugs versus placebo or not | Wichman, 2016 | 3 | 0 | 0 | 3 | 0 | serious risk | no serious  inconsistency | no serious  indirectness | no serious imprecision | NA | would not  reduce effect | no | no | low |
| TPOAb(9 month) | Combination of selenium and drugs versus placebo or not | Wichman, 2016 | 1 | 0 | 0 | 1 | 0 | serious risk | no serious  inconsistency | no serious  indirectness | no serious imprecision | NA | would not  reduce effect | no | no | low |
| TSH | Combination of selenium and drugs versus placebo or not | Zuo, 2021 | 8 | 0 | 0 | 8 | 0 | serious risk | serious  inconsistency | no serious  indirectness | no serious imprecision | strongly suspected | would reduce effect | no | no | very low |
| TGAb(3 month) | Combination of selenium and drugs versus placebo or not | Wichman, 2016 | 6 | 0 | 0 | 6 | 0 | serious risk | no serious  inconsistency | no serious  indirectness | no serious imprecision | NA | would not  reduce effect | no | no | low |
| TGAb(6 month) | Combination of selenium and drugs versus placebo or not | Wichman, 2016 | 3 | 0 | 0 | 3 | 0 | serious risk | no serious  inconsistency | no serious  indirectness | no serious imprecision | NA | would not  reduce effect | no | no | low |
| TGAb(9 month) | Combination of selenium and drugs versus placebo or not | Wichman, 2016 | 1 | 0 | 0 | 1 | 0 | serious risk | no serious  inconsistency | no serious  indirectness | no serious imprecision | NA | would not  reduce effect | no | no | low |
| ***Graves’ disease*** |  |  |  |  |  |  |  |  |  |  |  |  |  |  |  |  |
| FT3(3 months) | Combination of selenium and drugs versus placebo or not | Zheng, 2018 | 2 | 0 | 0 | 2 | 0 | serious risk | no serious  inconsistency | no serious  indirectness | no serious imprecision | undetected | would not  reduce effect | no | no | low |
| FT3(6 months) | Combination of selenium and drugs versus placebo or not | Zheng, 2018 | 4 | 0 | 0 | 4 | 0 | serious risk | no serious  inconsistency | no serious  indirectness | no serious imprecision | undetected | would not  reduce effect | no | no | low |
| FT3(9 months) | Combination of selenium and drugs versus placebo or not | Zheng, 2018 | 3 | 0 | 0 | 5 | 0 | serious risk | no serious  inconsistency | no serious  indirectness | no serious imprecision | undetected | would not  reduce effect | no | no | low |
| FT4(3 months) | Combination of selenium and drugs versus placebo or not | Zheng, 2018 | 2 | 0 | 0 | 2 | 0 | serious risk | no serious  inconsistency | no serious  indirectness | no serious imprecision | undetected | would not  reduce effect | no | no | low |
| FT4(6 months) | Combination of selenium and drugs versus placebo or not | Zheng, 2018 | 4 | 0 | 0 | 4 | 0 | serious risk | no serious  inconsistency | no serious  indirectness | no serious imprecision | undetected | would not  reduce effect | no | no | low |
| FT4(9 months) | Combination of selenium and drugs versus placebo or not | Zheng, 2018 | 3 | 0 | 0 | 5 | 0 | serious risk | no serious  inconsistency | no serious  indirectness | no serious imprecision | undetected | would not  reduce effect | no | no | low |
| TSH(3 months) | Combination of selenium and drugs versus placebo or not | Zheng, 2018 | 1 | 0 | 0 | 1 | 0 | serious risk | no serious  inconsistency | no serious  indirectness | no serious imprecision | undetected | would not  reduce effect | no | no | low |
| TSH(6 months) | Combination of selenium and drugs versus placebo or not | Zheng, 2018 | 3 | 0 | 0 | 3 | 0 | serious risk | no serious  inconsistency | no serious  indirectness | no serious imprecision | undetected | would not  reduce effect | no | no | low |
| TSH(9 months) | Combination of selenium and drugs versus placebo or not | Zheng, 2018 | 3 | 0 | 0 | 5 | 0 | serious risk | no serious  inconsistency | no serious  indirectness | no serious imprecision | undetected | would not  reduce effect | no | no | low |
| TRAb(6 months) | Combination of selenium and drugs versus placebo or not | Zheng, 2018 | 3 | 0 | 0 | 3 | 0 | serious risk | no serious  inconsistency | no serious  indirectness | no serious imprecision | undetected | would not  reduce effect | no | no | low |
| TRAb(9 months) | Combination of selenium and drugs versus placebo or not | Zheng, 2018 | 3 | 0 | 0 | 3 | 0 | serious risk | no serious  inconsistency | no serious  indirectness | no serious imprecision | undetected | would not  reduce effect | no | no | low |
| ***Hashimoto's thyroiditis*** |  |  |  |  |  |  |  |  |  |  |  |  |  |  |  |  |
| well-being/mood | ≤200 µg versus not | Toulis, 2010 | 3 | 0 | 0 | 3 | 0 | serious risk | serious  inconsistency | no serious  indirectness | no serious imprecision | undetected | Would reduce effect | no | no | very low |
| TPOAb | ≤200 µg versus not | Toulis, 2010 | 4 | 0 | 0 | 4 | 0 | serious risk | serious  inconsistency | no serious  indirectness | no serious imprecision | undetected | Would reduce effect | no | no | very low |
| ***Type 2 diabetes*** |  |  |  |  |  |  |  |  |  |  |  |  |  |  |  |  |
| incidence | 200 μg per day versus not | Vinceti, 2018 | 5 | 0 | 0 | 5 | 0 | serious risk | no serious  inconsistency | no serious  indirectness | no serious imprecision | undetected | would not  reduce effect | no | no | low |
| incidence | 120μg/day versus 55μg/day | Vinceti, 2021 | 6 | 3 | 2 | 0 | 0 | serious risk | no serious  inconsistency | no serious  indirectness | no serious imprecision | undetected | would not  reduce effect | no | no | low |
| HOMA-B | Combination of selenium and drugs versus placebo or not | Gorabi, 2020 | 4 | 0 | 0 | 4 | 0 | serious risk | no serious  inconsistency | no serious  indirectness | no serious imprecision | undetected | would not  reduce effect | no | no | moderate |
| HOMA-IR | Combination of selenium and drugs versus placebo or not | Ouyang. 2022 | 10 | 0 | 0 | 10 | 0 | serious risk | serious  inconsistency | no serious  indirectness | serious imprecision | undetected | would not  reduce effect | no | no | very low |
| Insulin level | Combination of selenium and drugs versus placebo or not | Gorabi, 2020 | 9 | 0 | 0 | 9 | 0 | serious risk | no serious  inconsistency | no serious  indirectness | no serious imprecision | undetected | would not  reduce effect | no | no | moderate |
| QUIKI | Combination of selenium and drugs versus placebo or not | Gorabi, 2020 | 4 | 0 | 0 | 4 | 0 | serious risk | no serious  inconsistency | no serious  indirectness | no serious imprecision | undetected | would not  reduce effect | no | no | moderate |
| FPG | Combination of selenium and drugs versus placebo or not | Gorabi, 2020 | 9 | 0 | 0 | 9 | 0 | serious risk | no serious  inconsistency | no serious  indirectness | no serious imprecision | undetected | would not  reduce effect | no | no | moderate |
| HbA1c | Combination of selenium and drugs versus placebo or not | Gorabi, 2020 | 3 | 0 | 0 | 3 | 0 | serious risk | no serious  inconsistency | no serious  indirectness | no serious imprecision | undetected | would not  reduce effect | no | no | moderate |
| ***Metabolic Syndrome*** | Highest versus lowest dietary selenium | Ding, 2022 | 4 | 0 | 0 | 0 | 4 | serious risk | no serious  inconsistency | no serious  indirectness | no serious imprecision | undetected | would not  reduce effect | no | no | low |
| ***Mental and cognitive outcomes*** |  |  |  |  |  |  |  |  |  |  |  |  |  |  |  |  |
| total depression | Highest versus lowest dietary selenium | Ding, 2022 | 6 | 2 | 0 | 0 | 4 | serious risk | no serious  inconsistency | no serious  indirectness | no serious imprecision | undetected | would not  reduce effect | no | no | low |
| Postpartum depression | Highest versus lowest supplementary selenium | Sajjadi, 2022 | 2 | 1 | 1 | 0 | 0 | serious risk | no serious  inconsistency | no serious  indirectness | no serious imprecision | undetected | would not  reduce effect | no | no | low |
| Depression scores | Highest versus lowest supplementary selenium | Sajjadi, 2022 | 3 | 0 | 0 | 2 | 1 | serious risk | no serious  inconsistency | serious  indirectness | no serious imprecision | undetected | would not  reduce effect | no | no | low |
| Other types of depression | Highest versus lowest supplementary selenium | Sajjadi, 2022 | 5 | 1 | 1 | 0 | 3 | serious risk | no serious  inconsistency | serious  indirectness | no serious imprecision | undetected | would not  reduce effect | no | no | low |
| ***Reproductive outcomes*** |  |  |  |  |  |  |  |  |  |  |  |  |  |  |  |  |
| ***Male Infertility*** |  |  |  |  |  |  |  |  |  |  |  |  |  |  |  |  |
| Sperm concentration | Combination of selenium and drugs versus placebo or not | Sharma, 2022 | 3 | 0 | 0 | 3 | 0 | serious risk | No serious  inconsistency | no serious  indirectness | no serious imprecision | NA | would not  reduce effect | no | no | low |
| Semen Volume | Combination of selenium and drugs versus placebo or not | Sharma, 2022 | 1 | 0 | 0 | 1 | 0 | serious risk | no serious  inconsistency | no serious  indirectness | no serious imprecision | NA | would not  reduce effect | no | no | low |
| Sperm motility | ≤200 µg versus not | Salas-Huetos, 2018 | 3 | 0 | 0 | 3 | 0 | serious risk | no serious  inconsistency | no serious  indirectness | no serious imprecision | undetected | would not  reduce effect | no | no | low |
| Sperm morphology | ≤200 µg versus not | Salas-Huetos, 2018 | 2 | 0 | 0 | 2 | 0 | serious risk | no serious  inconsistency | no serious  indirectness | no serious imprecision | undetected | would not  reduce effect | no | no | low |
| Pregnancy rate | Combination of selenium and drugs versus placebo or not | Sharma, 2022 | 1 | 0 | 0 | 1 | 0 | serious risk | no serious  inconsistency | no serious  indirectness | serious imprecision | NA | would not  reduce effect | no | no | very low |
| ***Polycystic ovary syndrome*** |  |  |  |  |  |  |  |  |  |  |  |  |  |  |  |  |
| SHBG | ≤200 µg versus not | Zhao, 2023 | 3 | 0 | 0 | 3 | 0 | serious risk | serious  inconsistency | no serious  indirectness | serious imprecision | undetected | would reduce effect | no | no | very low |
| total testosterone | ≤200 µg versus not | Wu, 2022 | 5 | 0 | 0 | 5 | 0 | serious risk | serious  inconsistency | no serious  indirectness | serious imprecision | undetected | would not reduce effect | no | no | very low |
| cholesterol | ≤200 µg versus not | Wu, 2022 | 5 | 0 | 0 | 5 | 0 | serious risk | serious  inconsistency | no serious  indirectness | serious imprecision | undetected | would not reduce effect | no | no | very low |
| ***Circulatory outcomes*** |  |  |  |  |  |  |  |  |  |  |  |  |  |  |  |  |
| ***Coronary heart disease*** |  |  |  |  |  |  |  |  |  |  |  |  |  |  |  |  |
| TC | Combination of selenium and drugs versus placebo or not | Kelishadi, 2022 | 19 | 0 | 0 | 19 | 0 | serious risk | no serious  inconsistency | no serious  indirectness | no serious imprecision | undetected | would not  reduce effect | no | no | moderate |
| VLDL-C | Combination of selenium and drugs versus placebo or not | Kelishadi, 2022 | 8 | 0 | 0 | 8 | 0 | serious risk | no serious  inconsistency | no serious  indirectness | no serious imprecision | undetected | would not  reduce effect | no | no | moderate |
| SBP | Combination of selenium and drugs versus placebo or not | Kelishadi, 2022 | 5 | 0 | 0 | 5 | 0 | serious risk | no serious  inconsistency | no serious  indirectness | no serious imprecision | undetected | would reduce effect | no | no | low |
| Mortality | Combination of selenium and drugs versus placebo or not | Ju, 2017 | 8 | 0 | 0 | 8 | 0 | serious risk | no serious  inconsistency | no serious  indirectness | no serious imprecision | undetected | would not  reduce effect | no | no | moderate |
| All CVD events | Combination of selenium and drugs versus placebo or not | Rees, 2013 | 2 | 0 | 0 | 2 | 0 | serious risk | no serious  inconsistency | no serious  indirectness | no serious imprecision | undetected | would not  reduce effect | no | no | moderate |
| TG | Combination of selenium and drugs versus placebo or not | Kelishadi, 2022 | 19 | 0 | 0 | 19 | 0 | serious risk | no serious  inconsistency | no serious  indirectness | no serious imprecision | undetected | would not  reduce effect | no | no | moderate |
| LDL-C | Combination of selenium and drugs versus placebo or not | Kelishadi, 2022 | 17 | 0 | 0 | 17 | 0 | serious risk | no serious  inconsistency | no serious  indirectness | no serious imprecision | undetected | would not  reduce effect | no | no | moderate |
| HDL-C | Combination of selenium and drugs versus placebo or not | Kelishadi, 2022 | 21 | 0 | 0 | 17 | 0 | serious risk | no serious  inconsistency | no serious  indirectness | no serious imprecision | undetected | would reduce effect | no | no | low |
| DBP | Combination of selenium and drugs versus placebo or not | Kelishadi, 2022 | 5 | 0 | 0 | 5 | 0 | serious risk | no serious  inconsistency | no serious  indirectness | no serious imprecision | undetected | would reduce effect | no | no | low |
| BMI | Combination of selenium and drugs versus placebo or not | Kelishadi, 2022 | 8 | 0 | 0 | 8 | 0 | serious risk | no serious  inconsistency | no serious  indirectness | no serious imprecision | undetected | would reduce effect | no | no | low |
| ***Keshan Disease*** | ever use versus not | Zhou, 2018 | 17 | NA | NA | 0 | 0 | serious risk | no serious  inconsistency | no serious  indirectness | no serious imprecision | NA | would not reduce effect | no | no | moderate |
| ***Skeletel outcomes*** |  |  |  |  |  |  |  |  |  |  |  |  |  |  |  |  |
| ***Kashin-Beck disease*** |  |  |  |  |  |  |  |  |  |  |  |  |  |  |  |  |
| Radiographic improvement | Combination of selenium and drugs versus placebo or not | Zou, 2019 | 22 | 0 | 0 | 22 | 0 | serious risk | no serious  inconsistency | no serious  indirectness | serious imprecision | undetected | would reduce effect | no | no | low |
| Risk | Combination of selenium and drugs versus placebo or not | Zou, 2008 | 4 | 0 | 0 | 4 | 0 | serious risk | no serious  inconsistency | serious  indirectness | serious imprecision | undetected | Would reduce effect | no | no | very low |
| ***Critical-ill outcomes*** |  |  |  |  |  |  |  |  |  |  |  |  |  |  |  |  |
| Total mortality | highest versus lowest | Manzanares, 2016 | 21 | 0 | 0 | 21 | 0 | serious risk | serious  inconsistency | no serious  indirectness | serious imprecision | undetected | would not  reduce effect | no | no | very low |
| New infectious complications | highest versus lowest | Manzanares, 2016 | 4 | 0 | 0 | 4 | 0 | serious risk | serious  inconsistency | no serious  indirectness | serious imprecision | undetected | would not  reduce effect | no | no | very low |
| Hospital stay | highest versus lowest | Mousavi, 2021 | 8 | 0 | 0 | 8 | 0 | serious risk | serious  inconsistency | no serious  indirectness | serious imprecision | undetected | would not  reduce effect | no | no | very low |
| ICU stay | highest versus lowest | Mousavi, 2021 | 11 | 0 | 0 | 11 | 0 | serious risk | serious  inconsistency | no serious  indirectness | serious imprecision | undetected | would not  reduce effect | no | no | very low |
| New renal dysfunction | highest versus lowest | Manzanares, 2016 | 10 | 0 | 0 | 10 | 0 | serious risk | serious  inconsistency | no serious  indirectness | serious imprecision | undetected | would not  reduce effect | no | no | very low |
| ***Survival*** |  |  |  |  |  |  |  |  |  |  |  |  |  |  |  |  |
| 28 day | highest versus lowest | Mousavi, 2021 | 19 | 0 | 0 | 19 | 0 | serious risk | serious  inconsistency | no serious  indirectness | serious imprecision | undetected | would not  reduce effect | no | no | very low |
| 3 months | highest versus lowest | Mousavi, 2021 | 3 | 0 | 0 | 3 | 0 | serious risk | serious  inconsistency | no serious  indirectness | serious imprecision | undetected | would not  reduce effect | no | no | very low |
| 6 months | highest versus lowest | Mousavi, 2021 | 2 | 0 | 0 | 2 | 0 | serious risk | serious  inconsistency | no serious  indirectness | serious imprecision | undetected | would not  reduce effect | no | no | very low |
| Ventilator days | highest versus lowest | Manzanares, 2016 | 8 | 0 | 0 | 8 | 0 | serious risk | serious  inconsistency | no serious  indirectness | serious imprecision | undetected | would not  reduce effect | no | no | very low |
| ***Infective outcomes*** |  |  |  |  |  |  |  |  |  |  |  |  |  |  |  |  |
| ***Spesis*** |  |  |  |  |  |  |  |  |  |  |  |  |  |  |  |  |
| Duration of vasopressor therapy | highest versus lowest | Li, 2019 | 4 | 0 | 0 | 4 | 0 | serious risk | serious  inconsistency | no serious  indirectness | serious imprecision | undetected | would not  reduce effect | no | no | very low |
| Respiratory tract infections | highest versus lowest | Nazari, 2022 | 1 | 0 | 1 | 0 | 0 | serious risk | serious  inconsistency | serious  indirectness | serious imprecision | NA | would reduce effect | no | no | very low |
| ***Mortality*** |  |  |  |  |  |  |  |  |  |  |  |  |  |  |  |  |
| 28 days | highest versus lowest | Li, 2019 | 13 | 0 | 0 | 13 | 0 | serious risk | no serious  inconsistency | no serious  indirectness | no serious imprecision | undetected | would not  reduce effect | no | no | low |
| 3 months | highest versus lowest | Li, 2019 | 2 | 0 | 0 | 2 | 0 | serious risk | no serious  inconsistency | no serious  indirectness | no serious imprecision | undetected | would not  reduce effect | no | no | low |
| 6 months | highest versus lowest | Li, 2019 | 1 | 0 | 0 | 1 | 0 | serious risk | no serious  inconsistency | no serious  indirectness | no serious imprecision | undetected | would not  reduce effect | no | no | low |
| New renal dysfunction | highest versus lowest | Li, 2019 | 5 | 0 | 0 | 5 | 0 | serious risk | no serious  inconsistency | no serious  indirectness | no serious imprecision | NA | would not  reduce effect | no | no | low |
| Secondary infection | highest versus lowest | Li, 2019 | 5 | 0 | 0 | 5 | 0 | serious risk | no serious  inconsistency | no serious  indirectness | no serious imprecision | NA | would not  reduce effect | no | no | low |
| ***Inflammatory markers*** |  |  |  |  |  |  |  |  |  |  |  |  |  |  |  |  |
| hs-CRP | highest versus lowest | Djalalinia, 2021 | 5 | 0 | 0 | 5 | 0 | serious risk | no serious  inconsistency | no serious  indirectness | serious imprecision | undetected | would not  reduce effect | no | no | low |
| CRP | highest versus lowest | Djalalinia, 2021 | 2 | 0 | 0 | 2 | 0 | serious risk | no serious  inconsistency | no serious  indirectness | serious imprecision | undetected | would not  reduce effect | no | no | low |
| NO | highest versus lowest | Djalalinia, 2021 | 4 | 0 | 0 | 4 | 0 | serious risk | no serious  inconsistency | no serious  indirectness | serious imprecision | undetected | would not  reduce effect | no | no | low |
| ***Other outcomes*** |  |  |  |  |  |  |  |  |  |  |  |  |  |  |  |  |
| Preeclampsia | 60 µg or 100 µg supplementation versus not | Xu, 2015 | 3 | 0 | 0 | 3 | 0 | serious risk | serious  inconsistency | no serious  indirectness | serious imprecision | undetected | would not  reduce effect | no | no | very low |
| ***Immune function*** |  |  |  |  |  |  |  |  |  |  |  |  |  |  |  |  |
| IgA | highest versus lowest | Filippini, 2022 | 3 | 0 | 0 | 3 | 0 | serious risk | no serious  inconsistency | no serious  indirectness | no serious imprecision | NA | would not  reduce effect | no | no | low |
| IgG | highest versus lowest | Filippini, 2022 | 2 | 0 | 0 | 2 | 0 | serious risk | no serious  inconsistency | no serious  indirectness | no serious imprecision | NA | would not  reduce effect | no | no | low |
| IgM | highest versus lowest | Filippini, 2022 | 2 | 0 | 0 | 2 | 0 | serious risk | no serious  inconsistency | no serious  indirectness | no serious imprecision | NA | would not  reduce effect | no | no | low |
| NK cells overall levels | highest versus lowest | Filippini, 2022 | 2 | 0 | 0 | 2 | 0 | serious risk | no serious  inconsistency | no serious  indirectness | no serious imprecision | NA | would not  reduce effect | no | no | low |
| lymphocyte overall levels | highest versus lowest | Filippini, 2022 | 4 | 0 | 0 | 4 | 0 | serious risk | no serious  inconsistency | no serious  indirectness | no serious imprecision | NA | would not  reduce effect | no | no | low |
| GRADE, Grading of Recommendations Assessment, Development, and Evaluation; RCT, randomized controlled trial; NRCT, non-randomized controlled trial; NA, not available. ATID, autoimmune thyroid disease; FT3, serum free triiodothyronine; FT4, Serum free thyroxine; TPOAb, anti-thyroid peroxidase antibody; TGAb, anti-thyroglobulin antibody; TRAb, thyrotrophic hormone receptor antibody; TSH, thyroid stimulating hormone; FPG, fasting plasma glucose; HbA1c, Hemoglobin A1c; HOMA-B, assessment-estimated β-cell function; QUICKI, quantitative insulin sensitivity check index; HOMA-IR, assessment-estimated insulin resistance; SHBG, sex hormone binding globulin.TC, total cholesterol; TG, triglyceride; CVD, cardiovascular disease; LDL-C, low-density lipoprotein-cholesterol; HDL-C, high-density lipoprotein-cholesterol; VLDL-C, very low density lipoprotein-cholesterol; DBP, diastolic blood pressure; SBP, systolic blood pressure; BMI, Body Mass Index; ICU, intensive care units; CRP, c-reactive protein; hs-CRP, high sensitivity c-reactive protein; NO, nitric oxide. IgA, immunoglobulin A; IgG, immunoglobulin G; IgM, immunoglobulin M, NK cells, natural kill cells. | | | | | | | | | | | | | | | | |
